# Identification of four novel T cell autoantigens and personal autoreactive profiles in multiple sclerosis

**DOI:** 10.1126/sciadv.abn1823

**Published:** 2022-04-27

**Authors:** Mattias Bronge, Klara Asplund Högelin, Olivia G. Thomas, Sabrina Ruhrmann, Claudia Carvalho-Queiroz, Ola B. Nilsson, Andreas Kaiser, Manuel Zeitelhofer, Erik Holmgren, Mathias Linnerbauer, Milena Z. Adzemovic, Cecilia Hellström, Ivan Jelcic, Hao Liu, Peter Nilsson, Jan Hillert, Lou Brundin, Katharina Fink, Ingrid Kockum, Katarina Tengvall, Roland Martin, Hanna Tegel, Torbjörn Gräslund, Faiez Al Nimer, André Ortlieb Guerreiro-Cacais, Mohsen Khademi, Guro Gafvelin, Tomas Olsson, Hans Grönlund

**Affiliations:** 1 Therapeutic Immune Design, Department of Clinical Neuroscience, Karolinska Institutet, Center for Molecular Medicine, 171 76 Stockholm, Sweden.; 2 Neuroimmunology Unit, Department of Clinical Neuroscience, Center for Molecular Medicine, Karolinska Institutet, 171 76 Stockholm, Sweden.; 3 Division of Vascular Biology, Department of Medical Biochemistry and Biophysics, Karolinska Institutet, 171 77 Stockholm, Sweden.; 4 Division of Affinity Proteomics, Department of Protein Science, SciLifeLab, KTH–Royal Institute of Technology, 171 65 Solna, Sweden.; 5 Neuroimmunology and MS Research Section (NIMS), Neurology Clinic, University of Zürich, University Hospital Zürich, 8091 Zürich, Switzerland.; 6 Department of Protein Science, KTH–Royal Institute of Technology, 114 21 Stockholm, Sweden.; 7 Department of Clinical Neuroscience, Division of Neurology, Karolinska Institutet, Karolinska University Hospital, 171 76 Stockholm, Sweden.; 8 Science for Life Laboratory, Department of Medical Biochemistry and Microbiology, Uppsala University, 752 37 Uppsala, Sweden.; 9 Human Protein Atlas, Department of Protein Science, KTH–Royal Institute of Technology, Stockholm, Sweden.

## Abstract

Multiple sclerosis (MS) is an inflammatory disease of the central nervous system (CNS), in which pathological T cells, likely autoimmune, play a key role. Despite its central importance, the autoantigen repertoire remains largely uncharacterized. Using a novel in vitro antigen delivery method combined with the Human Protein Atlas library, we screened for T cell autoreactivity against 63 CNS-expressed proteins. We identified four previously unreported autoantigens in MS: fatty acid–binding protein 7, prokineticin-2, reticulon-3, and synaptosomal-associated protein 91, which were verified to induce interferon-γ responses in MS in two cohorts. Autoreactive profiles were heterogeneous, and reactivity to several autoantigens was MS-selective. Autoreactive T cells were predominantly CD4^+^ and human leukocyte antigen–DR restricted. Mouse immunization induced antigen-specific responses and CNS leukocyte infiltration. This represents one of the largest systematic efforts to date in the search for MS autoantigens, demonstrates the heterogeneity of autoreactive profiles, and highlights promising targets for future diagnostic tools and immunomodulatory therapies in MS.

## Teaser

A broad screening of T cell reactivity identifies FABP7, PROK2, RTN3, and SNAP91 as novel CD4^+^ T cell autoantigens in MS.

## INTRODUCTION

Multiple sclerosis (MS) is an organ-specific inflammatory, likely autoimmune, disease of the central nervous system (CNS) in which cells of the adaptive immune system cross the blood-brain barrier (BBB) and cause localized CNS inflammation, demyelination, and axonal damage (*1*). While more common in certain brain regions and the spinal cord, the inflammation can occur practically anywhere within the CNS and gives rise to a wide variety of neurological impairments. A definite trigger for the disease has not yet been identified but is believed to depend on an interplay of genetic, environmental, and lifestyle factors, which leads to pathological immune responses (*2*). The human leukocyte antigen (HLA) class II DRB1 locus with a DR15 haplotype, carrying the DRB1*15: 01 and DRB5*01:01 alleles, is the strongest genetic risk factor for MS, with an approximately threefold increased risk of disease, and implicates a central role for CD4^+^ T cells in disease development (*3*).

Much effort has been put into identifying MS-relevant T cell autoantigens. While myelin proteins such as myelin basic protein (MBP), proteolipid protein (PLP), and myelin oligodendrocyte glycoprotein (MOG) are currently considered T cell targets in MS, data remain inconsistent (*4*–*11*). Several other candidates have been reported in recent years, notably RAS guanyl-releasing protein 2 (*12*), GDP-l-fucose synthase (*13*), β-synuclein (*14*), αB-crystalline (*15*), and the autoantibody target anoctamin 2 (*16*), but the autoantigen repertoire known so far is likely to be incomplete (*17*).

With the emergence of antigen-specific immunotherapies, the interest in identifying novel autoantigens has intensified alongside their potential as future treatment targets (*18*). This treatment strategy is hypothesized to be effective while safer than currently available broadly acting immune-modulatory agents associated with risks for side effects such as infections and possibly cancer (*19*, *20*). In animal models, where the autoantigens are both limited and defined, this strategy has shown promising results (*21*, *22*), whereas in clinical trials in MS treatments targeting myelin antigens, they have been, if safe, of modest efficacy at best (*23*–*27*). One explanation for the lack of efficacy is that effective tolerization therapy requires targeting of a greater number of autoantigens and preferably personalized according to each individual’s disease-driving autoantigens (*17*).

Identifying relevant autoantigens has proven difficult, especially since autoreactive T cells can be a part of the normal T cell repertoire, an observation that limits the usefulness of identifying rare T cell clones (*8*, *28*). In addition, the rarity of these cells renders comparisons of differences in frequencies or inflammatory characteristics challenging. Here, we used a sensitive FluoroSpot-based method (*29*, *30*) in combination with the vast protein epitope signature tag (PrEST) library from the Human Protein Atlas (HPA) project (*31*) to perform an extensive and broad screening of autoreactivity against CNS-expressed proteins with the aim of identifying previously unknown T cell targets in MS.

## RESULTS

### Broad T cell reactivity screening of patients with MS identifies fatty acid–binding protein 7, prokineticin-2, reticulon-3, and synaptosomal-associated protein 91 as candidate T cell autoantigens

**Fig. 1.**
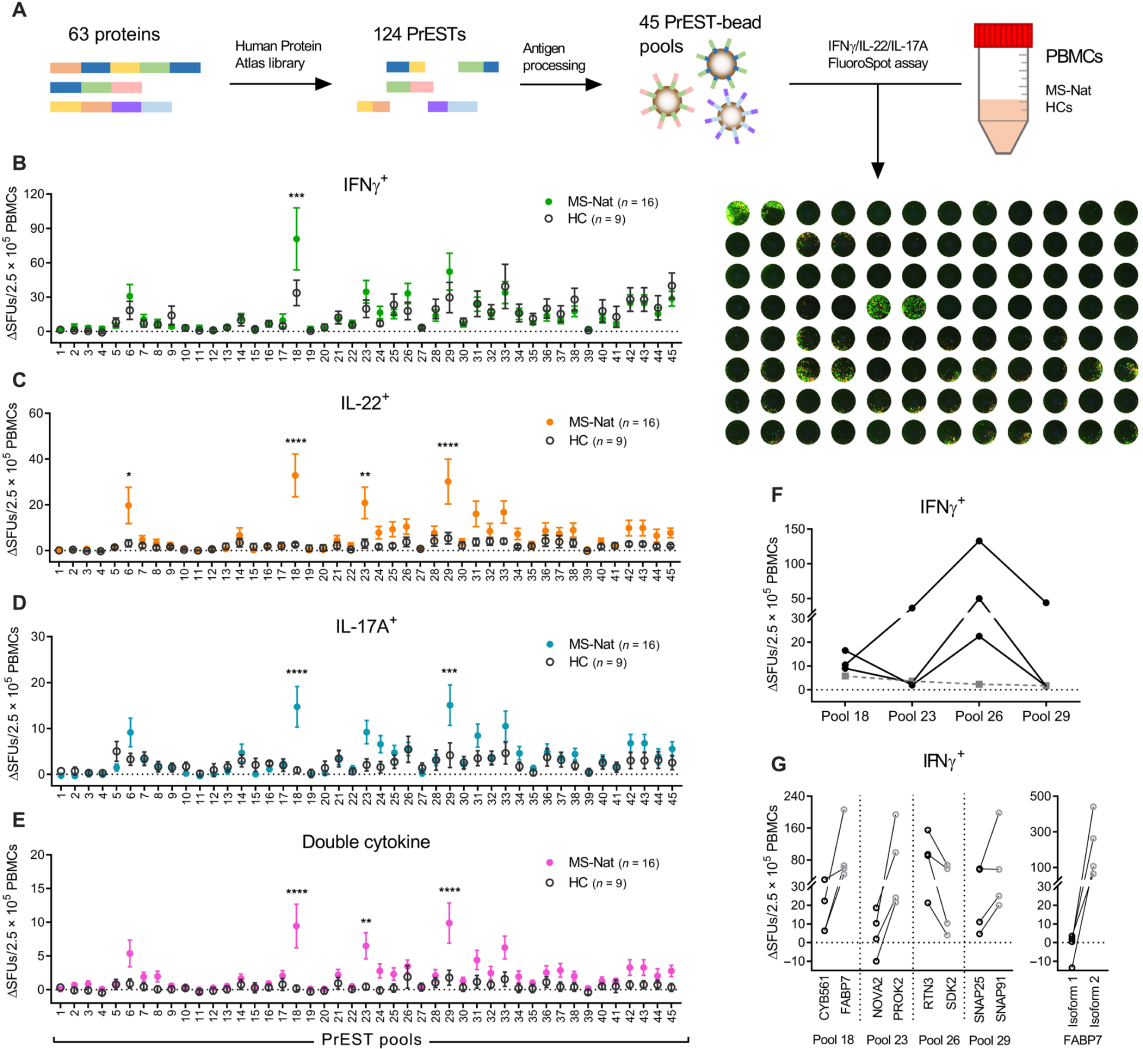
T cell reactivity screening using a PrEST library. A panel of PrESTs was tested for their ability to activate PBMCs from natalizumab-treated pwMSs (MS-Nat; *n* = 16) and age- and sex-matched HCs (*n* = 9) in an IFNγ/IL-22/IL-17A FluoroSpot assay. (**A**) Brief overview of the method with a representative developed FluoroSpot plate. (**B** to **E**) Number of spot-forming units (SFUs) above background response (ΔSFUs) per 2.5 × 10^5^ PBMCs producing (B) IFNγ, (C) IL-22, (D) IL-17A, or (E) two different cytokines in the FluoroSpot assay after stimulation with the PrESTs pools (#1 to #45). Means ± SEM presented for MS (closed, colored circles) and HCs (open, dark gray circles). *P* values were calculated with two-way analysis of variance (ANOVA) with Sidak correction for multiple comparisons. *P < 0.05, **P < 0.01, ***P < 0.001, and ****P < 0.0001. (**F**) IFNγ response in three pwMSs that had a high response to antigen pool #26. Black dots represent IFNγ spots with each line representing one individual and gray dashed line representing the three individuals’ mean IL-22 responses. (**G**) Four patients with MS with high responses to the PrEST pools in the screening were again tested with the PrESTs from the individual proteins contained in the pools (left graph). Four patients with responses against the FABP7 PrEST were also tested against recombinant full-length versions of the two different FABP7 isoforms (right graph). Each line represents one patient. CYB561, cytochrome b561; NOVA2, NOVA alternative splicing regulator 2; SDK2, sidekick cell adhesion molecule 2.

To identify novel autoantigens in MS, we mapped T cell reactivity against a panel of recombinant human PrESTs (*32*) from the HPA project (www.proteinatlas.org) (*31*). Antigens were chosen among proteins selectively expressed in the CNS but not previously described as antigens, established autoantigens, and previously studied candidate autoantigens. A total of 124 PrESTs from 63 different proteins were included (full list in table S1). The PrESTs were coupled to phagocytable particles, processed as previously described (*29*, *30*), and used to stimulate peripheral blood mononuclear cells (PBMCs) from natalizumab-treated persons with MS (pwMSs; *n* = 16) and age- and sex-matched healthy controls (HCs; *n* = 9) (cohort characteristics in table S2) in an interferon-γ (IFNγ)/interleukin-22 (IL-22)/IL-17A FluoroSpot assay. For the explorative screening, the PrESTs were mixed into 45 different pools (Fig. 1A).

While there were very similar responses between pwMSs and HCs for most PrESTs tested, a higher response in pwMSs was noted for four PrEST pools: #6, #18, #23, and #29 (Fig. 1, B to E). In addition, a higher mean IFNγ response was noted for pool #26, which was, while nonsignificant, in stark contrast to the rest of the screening panel (Fig. 1B). On closer look, there were three pwMSs displaying a strong and selective IFNγ response to pool #26 (Fig. 1F), and pool #26 was then also considered a positive hit in the screening. While pool #6 only contained PrESTs from the established autoantigen MOG, pools #18, #23, #26, and #29 included PrESTs from more than one protein. To determine the candidate autoantigens’ identity, we analyzed four patients with responses toward the pools with PrESTs from the individual proteins (Fig. 1G). In all cases, one of the proteins was responsible for most of the response: fatty acid–binding protein 7 (FABP7) from pool #18, prokineticin-2 (PROK2) from #23, reticulon-3 (RTN3) from #26, and synaptosomal-associated protein 91 (SNAP91) from #29. Four patients were also tested with recombinant full-length versions of the two isoforms of FABP7, showing no response to isoform 1 but a strong response to isoform 2 (Fig. 1G). Except for MOG, responses to other previously studied autoantigens were not increased in pwMSs. However, negative results do not exclude autoreactivity among these proteins due to a small cohort size and since crucial epitopes may potentially have been omitted in this explorative panel where only PrESTs and not full protein sequences were used.

### Confirmation of candidate autoantigens using full-length recombinant proteins

To validate findings from the explorative screening, full-length recombinant versions of the novel candidate autoantigens—as well as MOG, MBP, and PLP—were produced in-house. In addition, a negative control (NC) consisting of the recombinant protein purification tag and a cytomegalovirus (CMV) control antigen were produced. All antigens were processed as previously described (*30*). Full quality control of the autoantigen panel is presented in fig. S1 (A to E).

**Fig. 2.**
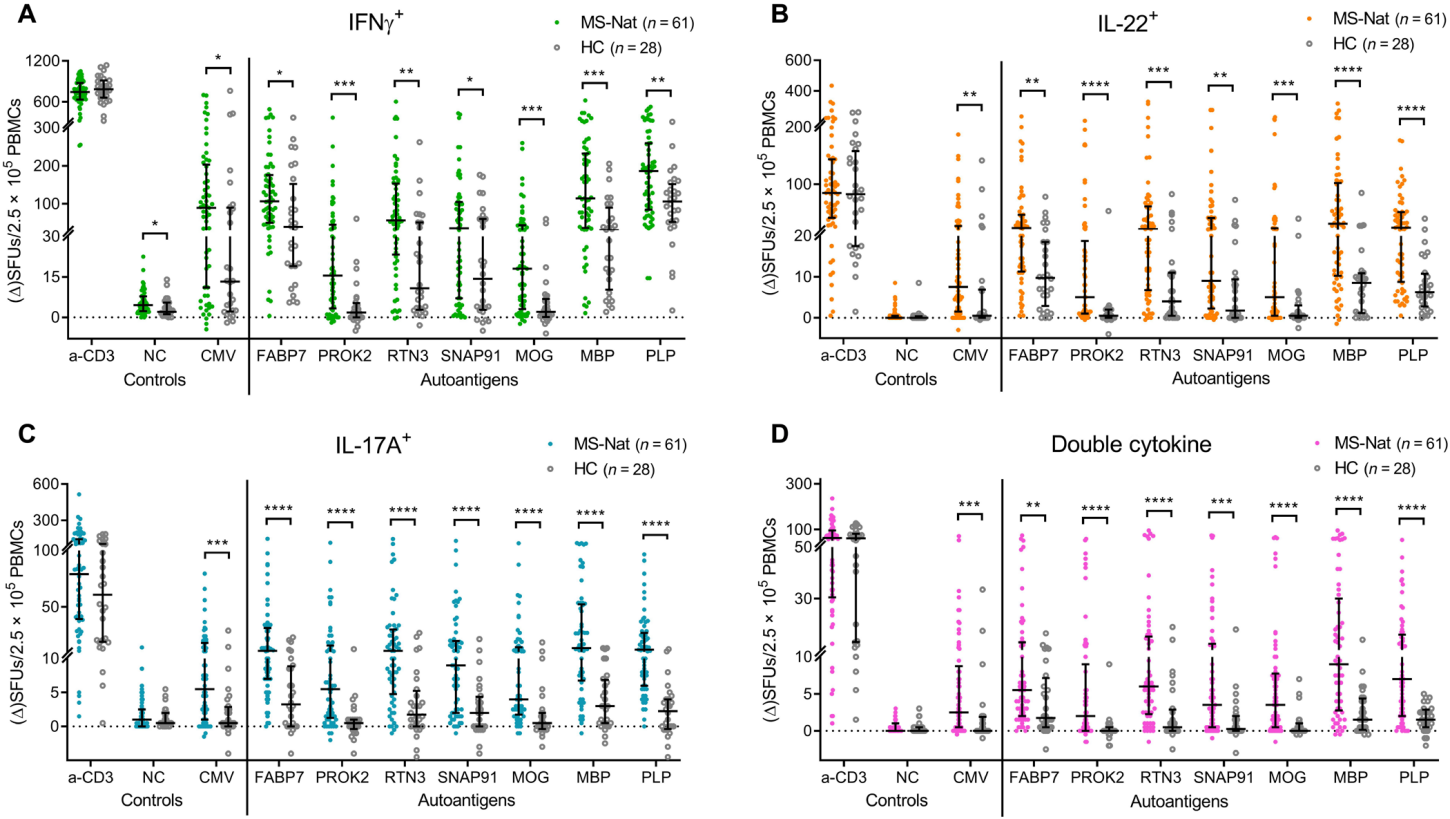
T cell reactivity using a full-length autoantigen panel. The four hits from the screening were produced as full-length proteins and were used as stimuli in an IFNγ/IL-22/IL-17A FluoroSpot assay, testing a cohort of natalizumab-treated pwMSs (*n* = 61) alongside age- and sex-matched HCs (*n* = 28). The number of SFUs producing (**A**) IFNγ, (**B**) IL-22, (**C**) IL-17A, or (**D**) two different cytokines after stimulation with the full-length autoantigen panel. Each dot represents one individual with brackets representing median and interquartile range (IQR). The anti-CD3 positive control stimulation is plotted as ΔSFUs per 1.25 × 10^5^ PBMCs, and NC is plotted as raw SFUs per 2.5 × 10^5^ PBMCs. *P* values were calculated with two-tailed Mann-Whitney *U* tests with false discovery rate (FDR) correction for multiple comparisons. FDR-adjusted *P* values are reported. *P < 0.05, **P < 0.01, ***P < 0.001, and ****P < 0.0001.

The full-length autoantigen panel was tested in a first validation cohort, consisting of natalizumab-treated pwMSs (*n* = 61) and age- and sex-matched HCs (*n* = 28), using an IFNγ/IL-22/IL-17A FluoroSpot. A significantly higher IFNγ response was observed in pwMSs for all autoantigens (Fig. 2A). Even larger differences were seen for IL-22 (Fig. 2B) and IL-17A (Fig. 2C) in natalizumab-treated pwMSs for all autoantigens, however, including for the CMV control. Similarly, double-cytokine responses were higher in the MS group in response to all autoantigens. Notably, autoantigen-specific IFNγ^+^IL-17^+^ and, to a lesser degree, IL-22^+^IL-17^+^ cells were selectively detected in pwMSs (fig. S2A).

To ensure that lipopolysaccharide (LPS) contamination did not bias the results, we performed correlation analyses between the antigen-bead LPS content and *P* values of the differences in responses. While antigens with higher endotoxin contamination displayed generally higher responses in all individuals, indicating higher background responses, the differences between pwMSs and HCs were not influenced, as there were no correlations for any cytokine (fig. S1E). There were also no differences in responses in the FluoroSpot for anti-CD3. However, the significantly higher CMV responses seen in pwMSs (Fig. 2, A to C), even if the difference was weaker than for the autoantigens, suggest that our method could be biased either because of the natalizumab treatment or because of some other factor inherent in MS. In conclusion, the first validation confirmed the findings of the screening for all autoantigens but raised questions regarding natalizumab-mediated influence.

### Autoreactivity and profiling in untreated pwMSs

**Fig. 3.**
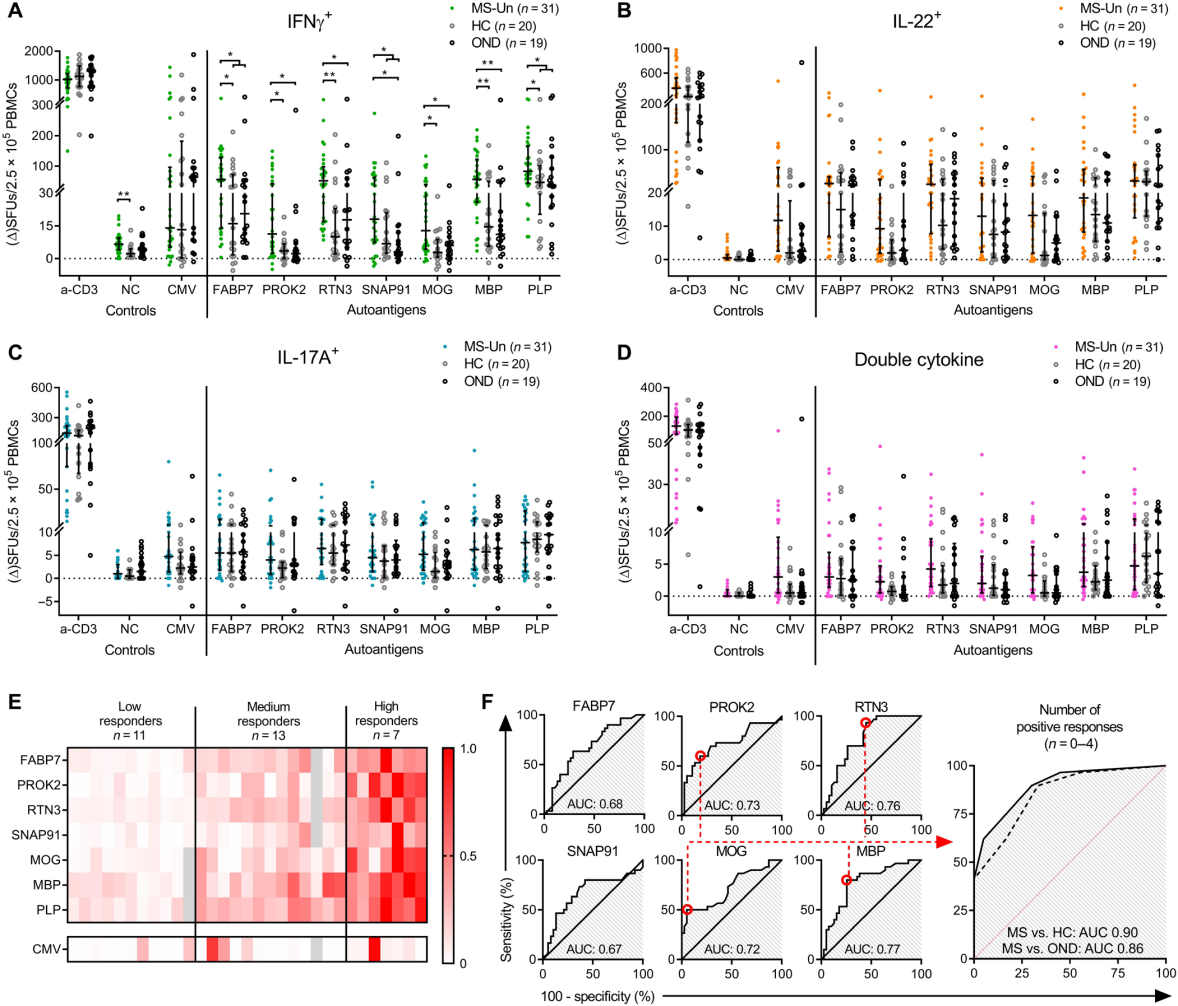
T cell reactivity and autoreactive profiles in untreated pwMSs. A second validation cohort consisting of untreated pwMSs (MS-Un) (*n* = 31), HCs (*n* = 20), and persons with OND (*n* = 19) was tested with the full-length autoantigen panel in a FluoroSpot assay. The number of SFUs producing (**A**) IFNγ, (**B**) IL-22, (**C**) IL-17A, or (**D**) two different cytokines after stimulation with the full-length autoantigen panel. Each dot represents one individual, and brackets represent median and IQR. The anti-CD3 positive control stimulation is plotted as ΔSFUs per 1.25 × 10^5^ PBMCs, and NC is plotted as raw SFUs. *P* values were calculated with two-tailed Mann-Whitney *U* tests with FDR correction for multiple comparisons. FDR-adjusted *P* values are reported. *P* values under 0.1 are shown. *P < 0.05 and **P < 0.01. (**E**) Heatmap of the individual IFNγ responses of pwMSs. Each column represents one individual. Intensity is based on the ratio of response/highest observed response for the same antigen (0.0 to 1.0). Grouped into low/medium/high responders based on the number of responses with a ratio of >0.25 (low = 0, medium = 1 to 5, and high = 6 to 7). Missing values are in gray. (**F**) ROC analysis to identify optimal cutoffs based on the IFNγ ΔSFUs of MS versus HC plus OND [data from graph in (A)]. The rightmost graph represents a composite test using the number of positive responses (*n* = 0 to 4), based on cutoffs for positivity of four autoantigens (PROK2, RTN3, MOG, and MBP) (red circles). The solid line represents MS versus HC, and the dashed line represents MS versus OND. AUC: 0.5 to 1.0, 0.5 = no predictive value and 1 = perfect test.

To eliminate a possible natalizumab effect, a second validation cohort was collected consisting of pwMSs that were untreated (*n* = 31), HCs (*n* = 20), and an additional control group consisting of persons with other neurological disease (OND) (*n* = 19). The higher IFNγ responses in pwMSs were confirmed when compared to each respective control cohort for PROK2, RTN3, MOG, and MBP (Fig. 3A). There were also increased responses toward FABP7 and SNAP91, but only significant compared to one of the control cohorts (FABP7 MS versus HC and SNAP91 MS versus OND), while the respective remaining groups showed a favorable trend: FABP7 MS versus OND (*P* = 0.079) and SNAP91 MS versus HCs (*P* = 0.112). Pooling the two control groups resulted in significantly higher responses in MS; adjusted *P* = 0.0272 for FABP7 pooled, compared to 0.039 and 0.079 for HC and OND independently, and adjusted *P* = 0.035 for SNAP91 pooled, compared to 0.112 and 0.035 for HC and OND. Similar results were seen for the classical MS autoantigen PLP (adjusted *P* = 0.0353 versus *P* = 0.048 and *P* = 0.1096). The complete statistical data are available in supplementary data files. No differences or trends thereof in IFNγ CMV responses were detected (*P* = 0.68 and *P* = 0.26 for HC and OND, respectively, and *P* = 0.68 for the pooled control group).

After correction for multiple comparisons, we could not detect significantly increased IL-22 or IL-17A responses in the untreated MS cohort (Fig. 3, B and C). Similar results were seen for double-cytokine–producing cells (Fig. 3D), which were of very low frequency (fig. S2B). In the pwMS versus OND comparison, there were positive correlations between LPS contamination and *P* values for IL-17A and double cytokines, meaning that, if anything, higher LPS and background responses led to smaller differences. No correlation was seen for IFNγ, IL-22, or in the MS versus HC comparison (fig. S1E).

The responses were heterogeneous, and individual patients displayed unique autoreactivity profiles, with different magnitudes of response and combinations of autoantigens. Overall, untreated pwMSs could be divided into low (*n* = 11), medium (*n* = 13), and high responders (*n* = 7) depending on the displayed number of autoreactive responses (zero to seven autoantigen targets) (Fig. 3E). To further explore the potential threshold for positivity and whether this autoantigen panel could serve as a diagnostic test, we performed receiver operating characteristic (ROC) tests based on the IFNγ response for all autoantigens (Fig. 3F), using pwMS versus the combined HC and OND control group. No single autoantigen proved particularly efficient in differentiating between MS and non-MS [area under curve (AUC) of 0.67 to 0.77]. Using the ROC tests to decide a positivity threshold, a composite test was made using positivity toward PROK2, RTN3, MOG, and MBP as readout (Fig. 3F). This test yielded an AUC of 0.90 [95% confidence interval (CI), 0.81 to 0.98; *P* < 0.0001] for MS versus HC and an AUC of 0.86 (95% CI, 0.75 to 0.96) for MS versus OND. Four (of four) positive responses resulted in a sensitivity of 41.4% (95% CI, 23.5 to 61.1%) at 100% specificity (95% CI, 83.2 to 100%/81.5 to 100% for MS versus HC/OND, respectively). One of four positive responses resulted in a sensitivity of 96.6% (95% CI, 82.2 to 99.9%) at a specificity of 55% (95% CI, 31.5 to 76.6%) and 44% (95% CI, 21.5 to 69.3%) for MS versus HC and OND, respectively. This test could be used to both confirm (four of four positives) and rule out (zero of four positives) an MS diagnosis with high accuracy.

### Characterization of the autoimmune response

**Fig. 4.**
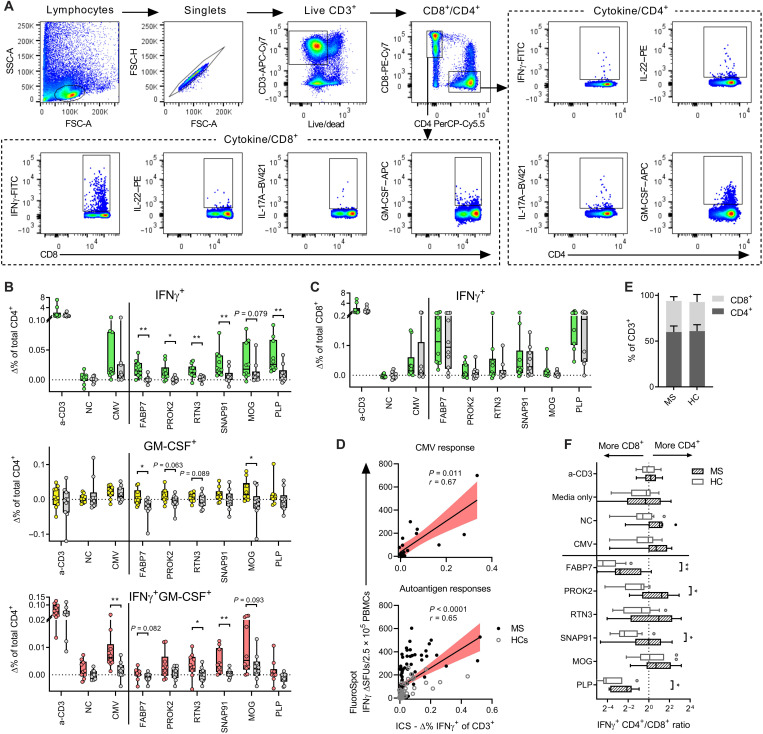
Cytokine profiling of autoantigen-specific T cells. Flow cytometry analysis of autoreactive T cells using intracellular cytokine staining after autoantigen stimulation. (**A**) Representative plots demonstrating gating strategy. SSC, side scatter; FSC; forward scatter. (**B**) IFNγ and GM-CSF responses in CD4^+^ T cells after stimulation with autoantigens or control antigens. (**C**) IFNγ responses in CD8^+^ T cells. (B) and (C) are plotted as the percentage of the total population after subtracting background responses (no stimuli, Δ%). Autoantigen-reactive pwMSs (*n* = 10) are in color, and HCs (*n* = 10) are in gray. Boxes represent median and IQR, and each dot represents one individual. *P* values were calculated using two-tailed Mann-Whitney *U* test and written as symbols when significant and in absolute numbers when 0.05 < *P* < 0.1. (**D**) Correlation between IFNγ SFUs as measured in FluoroSpot (*y* axis) and % IFNγ^+^ of total CD3^+^ population as measured with flow cytometry (*x* axis). The black line represents the linear regression slope, and the red area represents the 95% CI of the slope. *P* and *r* values were calculated using two-tailed nonparametric Spearman correlation. ICS, intracellular cytokine staining. (**E**) Proportion of CD4^+^ and CD8^+^ T cells in the CD3^+^ compartment of MS and HCs, based on unstimulated cells. Bars and staples represent means and SD. (**F**) Ratio of % IFNγ^+^CD4^+^ and % IFNγ^+^CD8^+^ after control and autoantigen stimulation in both MS and HCs. Boxes represent median and IQR, and brackets represent 1.5 × IQR (Tukey). *P* values were calculated using two-tailed Mann-Whitney *U* test and written when significant. For the whole figure, *P < 0.05 and **P < 0.01.

Cytokine responses toward FABP7, PROK2, RTN3, SNAP91, MOG, and PLP were further analyzed by flow cytometry. In addition to the cytokines included in the FluoroSpot analysis, we wanted to explore whether granulocyte-macrophage colony-stimulating factor (GM-CSF)–producing cells were targeting autoantigens specifically, as GM-CSF–producing CD4^+^ T cells have recently been demonstrated to be selectively increased in MS (*33*). Using intracellular cytokine staining after stimulation with autoantigens, CD4^+^ and CD8^+^ T cells were examined (Fig. 4A). PBMCs from autoantigen-responsive natalizumab-treated pwMSs (*n* = 10) were tested, and HCs (*n* = 10) were used as a comparison. Autoantigen-reactive IFNγ-producing CD4^+^ T cells were again detected in pwMSs with this second method (Fig. 4B), and, while the background responses of GM-CSF cells were generally high, there were significantly more GM-CSF–producing CD4^+^ cells in pwMSs after FABP7 and MOG stimulation, with a similar trend noted for PROK2 and RTN3. Coproducing IFNγ^+^GM-CSF^+^ CD4^+^ cells were similarly increased after CMV, RTN3, and SNAP91 stimulation. CD8^+^ T cells also responded with IFNγ production after autoantigen stimulation, but this response was not specific for pwMSs (Fig. 4C). The total IFNγ response of the CD3^+^ population strongly correlated with the same individuals’ FluoroSpot IFNγ spot-forming units (SFUs), both for CMV (*P* = 0.011) and autoantigens (*P* < 0.0001) (Fig. 4D), validating previous findings from the FluoroSpot assay. Similar to the FluoroSpot experiment, IFNγ^+^ IL-17A^+^ cells were selectively increased in pwMSs, while IL-22 and IL-17A responses did not differ between pwMSs and HCs in this experimental setup (fig. S3, A and B). Even if disregarding the magnitude of the response, pwMSs also displayed a higher IFNγ^+^ CD4^+^/CD8^+^ ratio after autoantigen stimulation when compared to HCs. In contrast, the control responses or general size of the CD4^+^ and CD8^+^ population was similar (Fig. 4, E and F).

### Influence of genetic and clinical factors on autoreactivity

**Fig. 5.**
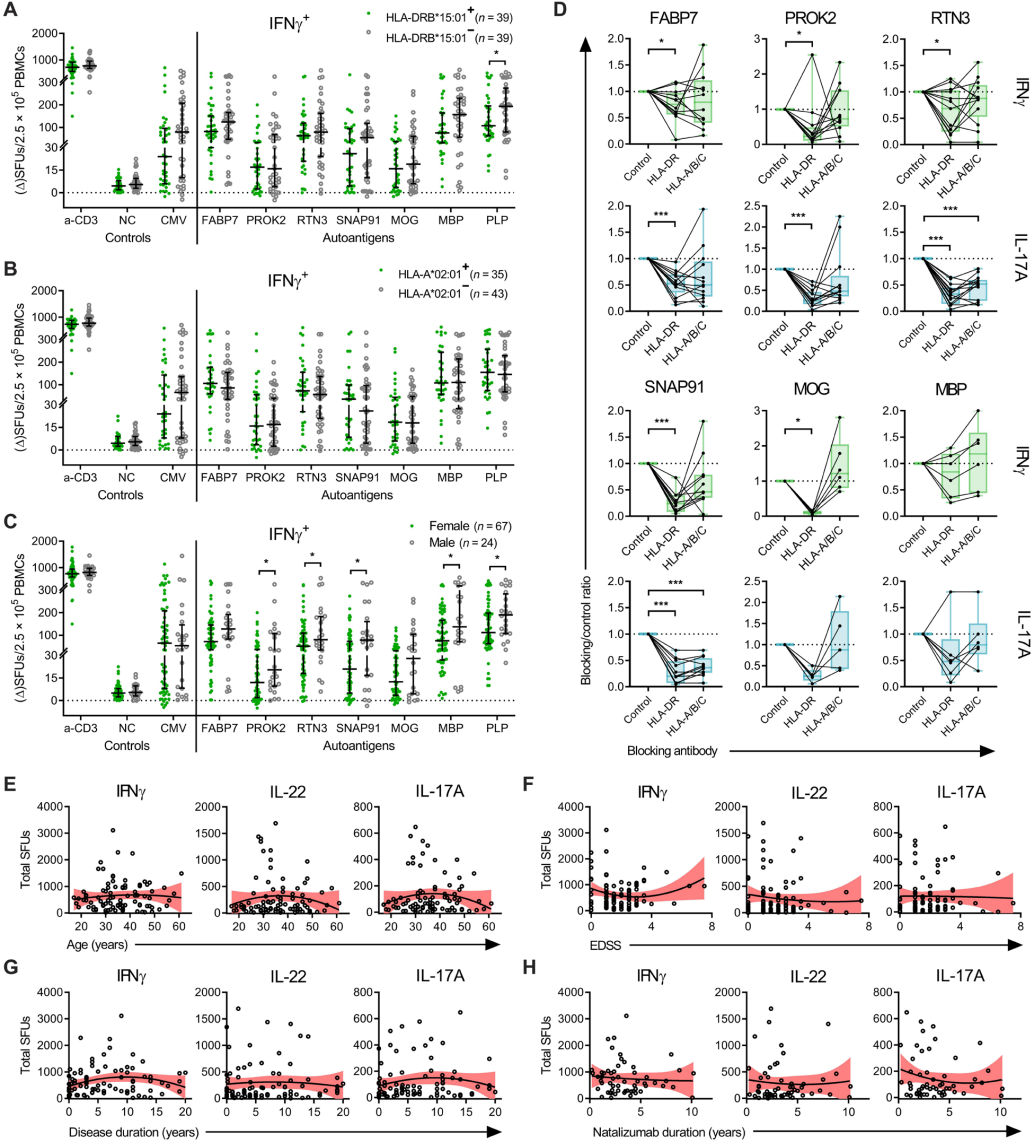
MHC and clinical correlations of autoreactivity. (**A** to **C**) Pooled IFNγ FluoroSpot results from the first and second validation cohort stratified on the basis of (A) HLA-DRB1*15:01 status, (B) HLA-A*02:01 status, and (C) sex. *P* values were calculated using two-tailed Mann-Whitney *U* test and shown when significant. (**D**) Effect of HLA blocking on autoantigen responses. MSs (*n* = 16, *n* = 6 for MBP) were stimulated with autoantigen together with HLA-DR or HLA-A/B/C blocking antibodies or an isotype control antibody. Unresponsive individuals (<6 ΔSFUs for IFNγ and <4 ΔSFUs for IL-17A) were excluded from the analysis. Results plotted as the ratio of blocking versus isotype control. Boxes represent median and IQR, with range in brackets. *P* values were calculated using two-tailed Wilcoxon signed-rank test. *P < 0.05 and ***P < 0.001. (**E** to **H**) Correlation between total autoantigen responses in the FluoroSpot assay of patients with MS and their clinical parameters (E) age, (F) expanded disability status scale (EDSS), (G) disease duration, and (H) natalizumab treatment duration. On the basis of pooled data from both the first and second validation cohort [except (H)]. *Y* axis represents the total sum of each individual’s autoantigen responses for the respective cytokines. Black lines represent the best-fit nonlinear regression slope, and the red area represents the 95% CI. *P* values were calculated using two-tailed nonparametric Spearman correlation tests, but none reached significance.

Next, we investigated whether clinical characteristics or the major MS risk gene HLA-DRB1*15:01 and protective gene HLA-A*02:01 (*34*) influenced the frequency of autoantigen-specific cells (Fig. 5, A to C). Stratification of autoantigen responses based on DRB1*15:01 and A*02:01 status did not reveal any major differences in responses between the groups (Fig. 5, A and B). While not reliant on DRB1*15:01 specifically, the epitope presentation and T cell activation were found to be dependent on HLA-DR, as HLA-DR–blocking antibodies removed a significant portion of both the IFNγ and IL-17A responses in the FluoroSpot assay (Fig. 5D). Moreover, while not as large of a reduction as observed for HLA-DR blocking, blocking HLA-A/B/C reduced some individuals’ responses, however, only significantly at a group level for IL-17A responses to RTN3 and SNAP91 (Fig. 5D).

There were higher autoantigen responses in males compared to females for PROK2 (*P* = 0.029), RTN3 (*P* = 0.049), SNAP91 (*P* = 0.023), MBP (*P* = 0.024), and PLP (*P* = 0.047), with a similar trend noted for FABP7 (*P* = 0.055) and MOG (*P* = 0.0971), while there was no difference in background, polyclonal, or CMV responses (*P* = 0.51, *P* = 0.41, and *P* = 0.98, respectively) (Fig. 5C). There were no significant correlations between total autoantigen responses and age, expanded disability status scale (EDSS), disease duration, or natalizumab treatment duration (Fig. 5, E to H). The strongest responses were seen in pwMSs in the early to mid-phase of the disease duration spectrum, with generally weaker responses in either recently diagnosed patients or those that had progressed further in their disease course (Fig. 5H). Nonetheless, persons with first known symptoms within 1 year of sampling did not have significantly lower responses to any of the specific autoantigens, suggesting that possible epitope spreading occurs before clinical onset (fig. S4). However, no definite conclusions could be drawn from this limited sample size (Fig. 5H).

### Presence of circulating autoantibodies

**Fig. 6.**
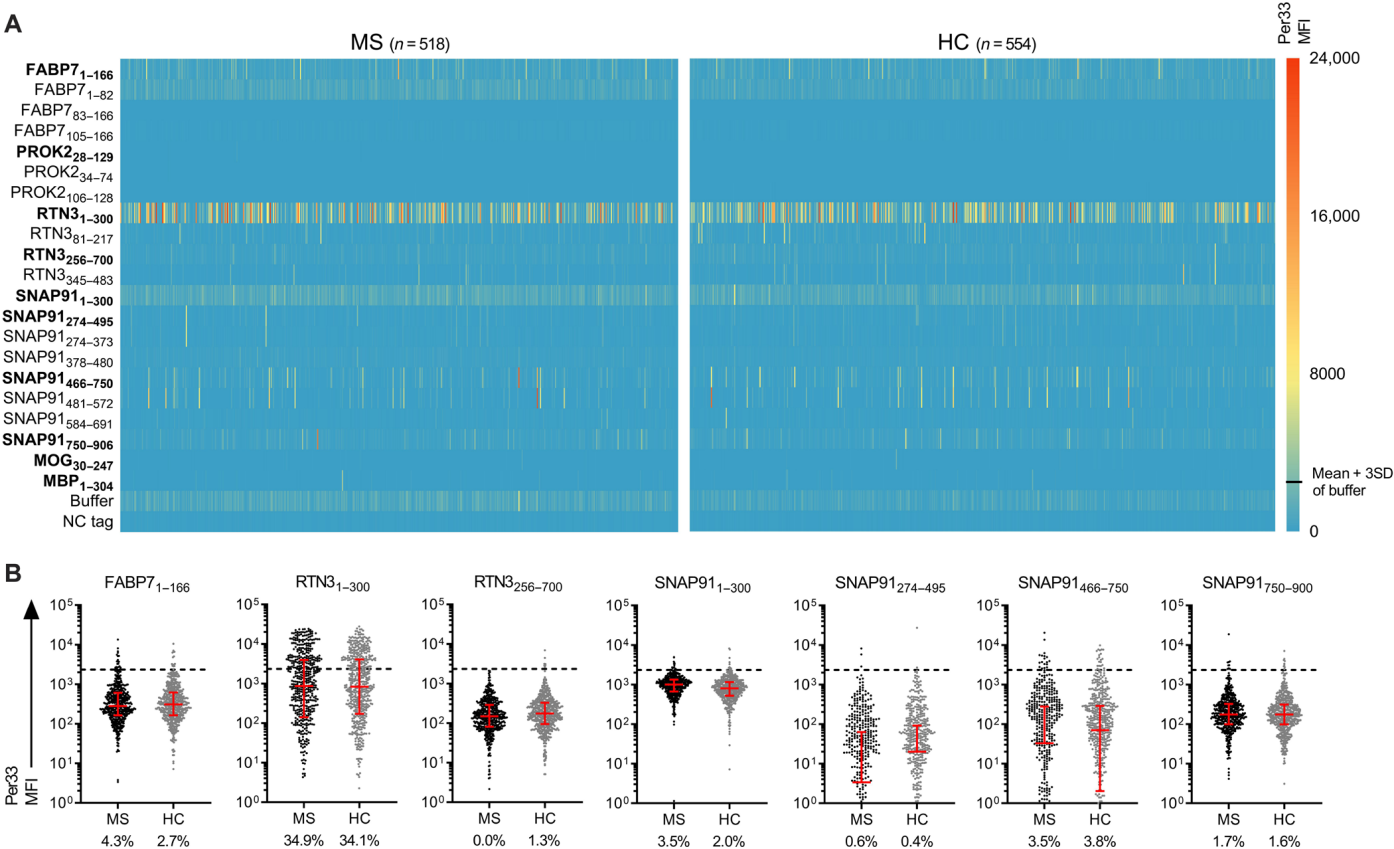
Autoantibodies targeting T cell autoantigens. The presence of autoantibodies was tested in a large cohort of pwMSs (*n* = 518) and HCs (*n* = 554) using a suspension bead array. Both the full-length autoantigens and PrESTs from the HPA of the corresponding proteins were used for detection. (**A**) Heatmap showing the adjusted antibody signal intensity, per33 median fluorescent intensity (MFI) of pwMSs (left heatmap) and HC (right heatmap) for the antigens. Each row represents one antigen, and each column represents one individual. Full-length antigens that were used in the FluoroSpot analysis are written in bold. (**B**) Dot plots showing the responses to the full-length antigens and frequencies of positive responses. The threshold for positivity (dashed line) was determined as the mean + 3SD of the response to the buffer (≥2350 MFI). Brackets represent median and IQR. *P* values were calculated using two-tailed Fisher’s exact test with Holm-Sidak correction for multiple comparisons.

To examine potential autoreactive antibodies targeting any of the identified autoantigens, we performed a suspension bead array, testing plasma from a large cohort of pwMSs (*n* = 518) and HCs (*n* = 554) (31 and 22, respectively, also tested for T cell reactivity in the FluoroSpot assay) (Fig. 6). There were frequent immunoglobulin G (IgG) responses to RTN3_1–300_ in both pwMSs and HCs, with positive responses in 181 of 518 (34.9%) and 189 of 554 (34.1%) individuals, respectively (odds ratio, 1.04; 95% CI, 0.81 to 1.33; *P* = 0.99) (Fig. 6, A and B). Other autoantibodies were, however, rare (0.0 to 5.0% positive individuals), with no differences between pwMSs and HCs (*P* > 0.05 in all cases). As responses to RTN3_81–217_ and RTN3_256–700_ were infrequent compared to RTN3_1–300_, we performed an in silico basic local alignment search tool for the RTN_1–300_ unique parts, including a 10–amino acid overlap with the nonreactive fragments (amino acids 1 to 90 and 207 to 266); however, no significant matches were found. There were no correlations between autoantibody responses to RTN3_1–300_ and T cell responses, either for IFNγ or IL-17A (fig. S5, A and B). The RTN3_1–300_ responses were independently confirmed using an enzyme-linked immunosorbent assay (fig. S5, C and D), using an alternative RTN_1–300_ version with a different purification tag, and the two methods strongly correlated (*r* = 0.79, *P* < 0.0001).

### Encephalitogenic potential of the novel autoantigens

**Fig. 7.**
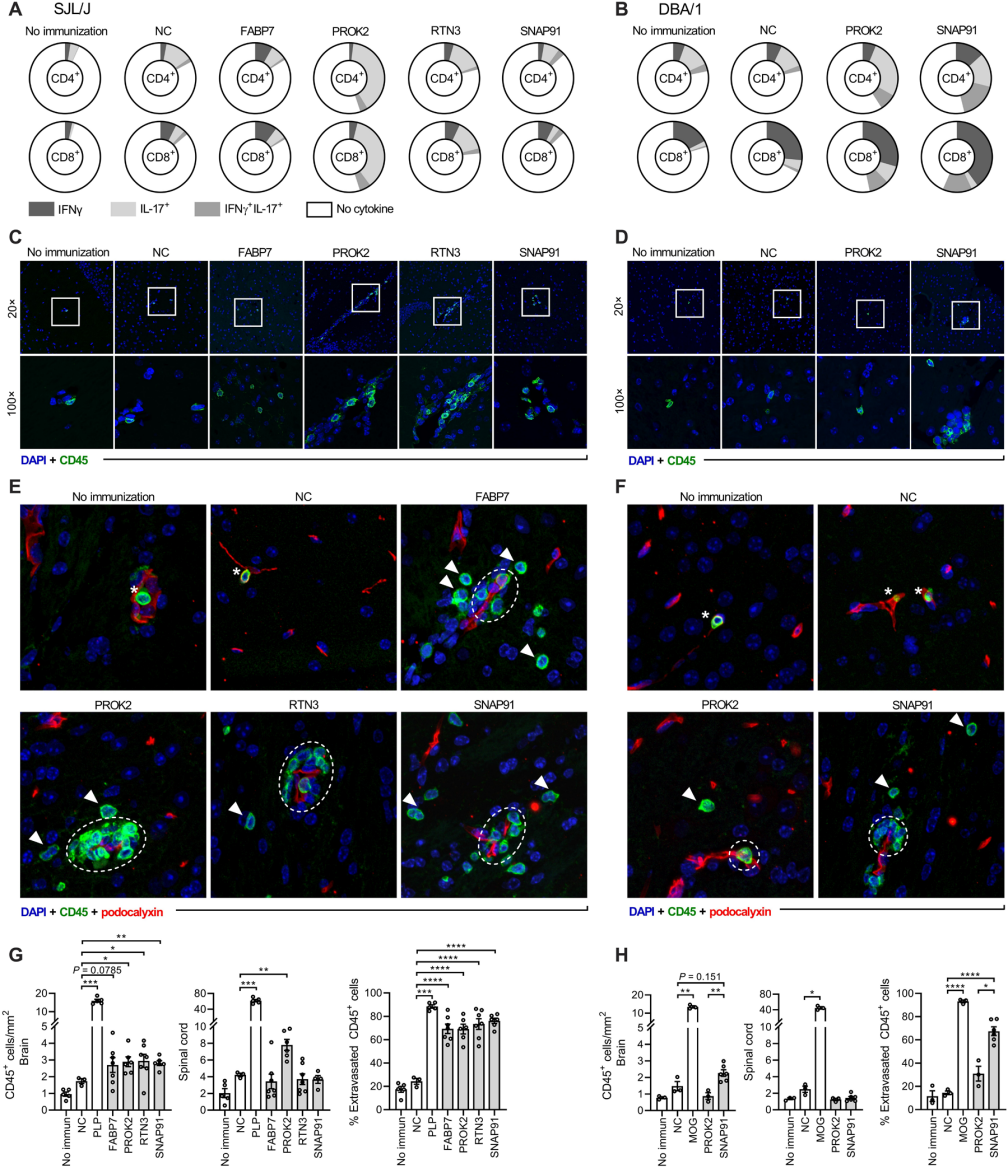
Encephalitogenic potential in mouse models. (**A**) Recall autoantigen responses in immunized SJL/J mice measured via intracellular cytokine staining and flow cytometry after ex vivo stimulation of splenocytes. (**B**) Recall responses in DBA/1 mice. Data shown represent the percentage of cytokine-positive CD4^+^/CD8^+^ cells (whole circle, 100% of cell population), displaying the mean results of all mice in the group (*n* = 3 to 8 per antigen) after stimulation with the same antigen as used for immunization. (**C** and **D**) Representative immunofluorescence of infiltrating CD45^+^ cells (green) in the brain of (C) SJL/J and (D) DBA/1 mice. Cell nuclei are visualized by 4′,6-diamidino-2-phenylindole (DAPI) (blue). Representative maximum intensity projections of 12-μm z-stacks are shown. White square in the 20× images indicates the area visible in 100×. (**E** and **F**) Representative immunofluorescence of the location of infiltrating CD45^+^ cells (green) in relation to blood vessels (red). Arrowheads indicate intraparenchymal leukocytes, dashed white circles indicate perivascular leukocytes, and asterisks indicate intravascular leukocytes. Full-size images are available in fig. S7. (**G**) Evaluation of CD45^+^ leukocyte infiltration into the brain and spinal cord in SJL/J mice after autoantigen immunization. Data from two experiments. No immunization (*n* = 5), NC (*n* = 3), PLP (*n* = 5), FABP7 (*n* = 7), PROK2 (*n* = 6), RTN3 (*n* = 7), and SNAP91 (*n* = 6). Rightmost graph shows the quantification of brain-infiltrating leukocytes that passed through the BBB. (**H**) Evaluation of CD45^+^ leukocyte infiltration into the brain and spinal cord of DBA/1 mice after autoantigen immunization. Data from one experiment. No immunization (*n* = 3), NC (*n* = 3), PLP (*n* = 3), PROK2 (*n* = 3), and SNAP91 (*n* = 6). Rightmost graph shows the quantification of brain-infiltrating leukocytes that passed through the BBB. Each dot represents one mouse, and bar and brackets represent means ± SEM. *P* values were calculated using one-way Brown-Forsythe and Welch ANOVA with Holm-Sidak correction for multiple comparison, comparing each group with the NC-immunized group. *P* values are shown when significant. *P < 0.05, **P < 0.01, **P < 0.001, and ****P < 0.0001.

The immunogenicity and encephalitogenic potential of the novel autoantigens were studied in a murine model of neuroinflammation. SJL/J and DBA/1 mice were immunized with the novel autoantigens or control antigens known to elicit a typical experimental autoimmune encephalomyelitis (EAE) disease course (PLP or MOG for SJL/J and DBA/1, respectively) together with adjuvants. Ex vivo analysis showed that all autoantigens induced antigen-specific T cell responses after immunization in the SJL/J strain, while only PROK2 and SNAP91 induced responses in the DBA/1 strain (Fig. 7, A and B, and fig. S6). We thus examined leukocyte CNS infiltration by immunofluorescence in mice with immune responses to the antigens, which revealed moderate infiltration of CD45^+^ cells in the brain of SJL/J mice after immunization with PROK2, RTN3, and SNAP91, with a similar trend noted for FABP7 (*P* = 0.0785), when compared to the mice immunized with the NC tag and adjuvant (Fig. 7, C and G). Both the infiltration and immune response patterns of individual antigens were heterogeneous. More specifically, PROK2 induced a strong IL-17 response in SJL/J mice (Fig. 7A) and led to both brain and spinal cord infiltration (Fig. 7G), while no infiltration was observed in the DBA/1 strain despite antigen-specific priming in the periphery (Fig. 7, D and H). In contrast, FABP7, RTN3, and SNAP91 had proportionally larger IFNγ^+^ and IFNγ^+^IL-17^+^ responses (Fig. 7A) and induced brain, but not spinal cord, infiltration in SJL/J mice (Fig. 7, C and G). SNAP91 elicited T cell responses, and there was a trend toward increased brain infiltration in DBA/1 mice compared to the NC immunization, while significantly increased compared to PROK2 (Fig. 7, B, D, and H). In all cases with autoantigen-induced leukocyte infiltration, the leukocytes had crossed the BBB, mainly infiltrating the perivascular space, while some had migrated deeper into the CNS parenchyma (Fig. 7, E to H, and fig. S7). In contrast, a majority of identified leukocytes in the control immunization failed to penetrate the BBB and were still located inside the blood vessel lumens. This implies that, in addition to CNS homing, there is a gain of pathological function in the autoantigen-primed cells. However, despite immunological responses and moderate CNS infiltration, none of the mice immunized with the four novel autoantigens exhibited clinical symptoms usually measured during EAE (tail weakness, limb paresis/paralysis, or balance disturbance) under the observation period of 40 days.

## DISCUSSION

In this study, we performed a broad screening of T cell reactivity against CNS proteins and identified FABP7, PROK2, RTN3, and SNAP91 as T cell–targeted autoantigens in MS. The findings of the explorative screening were confirmed in two validation cohorts using two methods. In particular, there was an increase in autoantigen-specific IFNγ-producing CD4^+^ T cells in the circulation of pwMSs. This is, to our knowledge, the first time that these four proteins have been identified as T cell targets in MS.

FABP7 is an intracellular protein involved in the transportation of hydrophobic ligands. It is selectively detected in CNS with low region specificity (HPA, www.proteinatlas.org/ENSG00000164434-FABP7), is primarily expressed in glial cells, and is increased in demyelinating regions in EAE (*35*). It has also been demonstrated that expression of FABP7 positively correlates with oligodendrocyte progenitor cells and remyelination, with decreased expression in the rim of chronic lesions (*36*). FABP7 is thus known to be present in areas of inflammation in MS, and FABP7 autoimmunity could potentially contribute to remyelination failure. Our data suggest that autoreactivity selectively targets the unique C terminus of isoform 2.

PROK2 is a secreted protein that regulates several different processes (*37*). It functions as an output molecule of the suprachiasmatic nucleus with ubiquitous receptor expression throughout the CNS and plays a role in the circadian rhythm (*38*). RNA expression is detected mainly in blood and bone marrow (HPA, www.proteinatlas.org/ENSG00000163421-PROK2). Circadian rhythm sleep disorders are commonly reported in MS and have been associated with increased fatigue (*39*).

RTN3 is a protein of the reticulon family, a group of membrane-bound proteins involved in protein transportation (*40*). RTN3 is mainly associated with the endoplasmic reticulum, with increased expression throughout the CNS, particularly in neuropil and neuronal cell bodies in gray matter (HPA, www.proteinatlas.org/ENSG00000133318-RTN3). While RTN3 is highly expressed in the CNS, it was recently shown to be present in plasma of pwMSs. This antigen has been described as a potential biomarker, as it decreased after treatment with natalizumab (*41*).

SNAP91, also named clathrin coat assembly protein AP 180, is a neuronal protein acting as a part of the vesicle formation system in synapses and is expressed throughout CNS gray matter, preferably within the neuropil because of the synaptic association (HPA, www.proteinatlas.org/ENSG00000065609-SNAP91) (*42*). Unlike more classic myelin-derived EAE antigens, neuronal gray matter antigens have recently been shown to induce more neurodegenerative features often found in MS but not in classical EAE (*14*). Hence, SNAP91 becomes an attractive new candidate for involvement in the neurodegenerative processes of MS.

Our methodology paves the way for the development of high-throughput assays testing numerous antigens in an unbiased manner. Still, the screening leaves room for improvement as PrESTs and not whole proteins were used, potentially omitting essential epitopes, and it was not sufficiently powered to identify rare T cell targets. Moreover, our strategy when designing the screening panel was to focus on CNS proteins. However, autoantigens are not necessarily exclusively or primarily expressed in the affected organ (*43*). Two recently identified MS autoantigens, RAS guanyl-releasing protein 2 (*12*) and GDP-l-fucose synthase (*13*), as well as the previously studied αB-crystalline, are not CNS specific. Hence, while our approach may increase the likelihood of finding autoantigens, one should still consider non-CNS antigens as potentially relevant targets as well. The 63–CNS protein panel in our screening, while extensive, did not encompass all CNS-enriched proteins and still contained four autoantigens, suggesting that the actual autoantigen repertoire might consist of several more.

In natalizumab-treated pwMSs, there was a notable increase in autoantigen-specific IL-22, IL-17A, and double-cytokine–producing cells, which was not seen in untreated pwMSs. As indicated by an increase in CMV responses, a general natalizumab effect was noted. However, it was less pronounced than for the autoantigen responses and, moreover, not directed toward CNS proteins in general, as seen in the responses to the screening panel. A direct natalizumab effect on T cells leading to increased cytokine expression is a possible explanation (*44*); however, it has been shown that this effect is not strong (*45*, *46*) and is further supported by the nonincrease in polyclonal responses. An argument can be made for false-negative results in untreated patients. As natalizumab blocks the integrin very late antigen-4 (VL4)–dependent T cell CNS migration (*47*), T cells that would otherwise infiltrate the CNS accumulate in venous blood. T helper 17 (T_H_17) cells are particularly migratory, which would lead to a larger proportional increase in these cells (*48*). Natalizumab also blocks migration to the gut, and gut microbiota can prime T_H_17 cells that later traffic to the CNS (*49*), and cross-reactivity between the gut bacteria *Akkermansia* and CNS autoantigens has recently been reported (*3*). This fits with our observations, as only antigen-specific T_H_17 responses, and not polyclonal or background, were increased. The high T_H_17 responses are likely biologically relevant and possibly more pronounced in the CNS compartment. Ethical considerations made this question difficult to fully resolve, as it would require treating healthy individuals with natalizumab, and we did not have access to a natalizumab-treated disease control group. Natalizumab treatment might be both a limitation and an advantage, as it could increase the chances to detect autoreactive cells usually only present in the CNS. Still, the usage of peripheral cells is a limitation in our study, as it may incompletely reflect what happens in the CNS. However, the low number of cells in CSF does not lend itself to a screening approach similar to this.

It has recently been shown by Galli *et al.* (*33*) that GM-CSF–producing CD4^+^ T cells are selectively increased in MS. Our findings expand upon this as we demonstrated the presence of autoantigen-specific GM-CSF CD4^+^ T cells in pwMSs, suggesting that the cell population reported by Galli *et al.* (*33*) are not only MS specific but also autoreactive and hence likely pathogenic. While CD4^+^ T cells have been implicated as the most important cell population in MS, our results from the flow cytometry and HLA-blocking experiments point toward a substantial autoreactive CD8^+^ population. This is not entirely unexpected as myelin-reactive CD8^+^ T cells have been described previously (*50*–*52*). However, there are indications that CD8^+^ T cells may perform a more regulatory role in MS (*53*), which may account for the more frequent autoreactive CD8^+^ than CD4^+^ T cells observed in healthy individuals in our study.

We screened a large cohort for potential autoantibodies, and no increase was detected in pwMSs. Therefore, we would like to argue that it is unlikely that antibody responses to any of these autoantigens play a distinct role in MS pathogenesis. Similarly, while MOG-specific T cells are increased in MS, anti-MOG antibodies are associated with distinctly different neuroinflammatory diseases and are rare in MS (*29*, *54*). However, while we used the long recombinant versions and PrESTs, which were designed for antibody recognition, it is possible that the proteins were not natively folded and, hence, conformationally dependent autoantibodies could be missed. Still, B cells’ role in MS seems to be antigen presentation and regulation of T cell responses rather than antibody production (*12*, *55*). It has been found that even on a genetic level, MS is distinct and more T cell oriented than autoimmune diseases where autoantibodies are common (*56*).

To explore encephalitogenic potential, we immunized two different mouse strains with the four novel autoantigens. Although no classical clinical signs of EAE were observed, post-euthanasia analyses revealed CNS leukocyte infiltration through the BBB in response to all autoantigens, indicating that they are encephalitogenic. The antigens showed different patterns of infiltration depending on the mouse strain, indicating that there is an influence of the major histocompatibility complex (MHC) background on the potential of the autoantigens to be encephalitogenic. The absence of a typical EAE phenotype is not unexpected since standard immunization leading to motor impairment is usually achieved with myelin antigens such as MOG, MBP, and PLP. In contrast, the novel autoantigens are of glial and neuronal origin. Even in some myelin antigen–based models, extensive infiltration can occur in animals that appear and behave completely healthy (*57*). SJL/J and DBA/1 were chosen since the break of tolerance against myelin antigens in these strains requires mild immunization protocols. SJL/J and DBA/1 also differ in their MHC haplotypes (H2s and H2q, respectively), both different from more standard strains extensively used in EAE experiments such as C57BL/6 (H2b haplotype). As both MHC and non-MHC genetic background influence the susceptibility of EAE for different antigens, stronger CNS inflammation could possibly occur in alternative strains or species (*58*). In addition, human versions of the proteins were applied, which could reduce or even completely preclude self-recognition; however, the homology between the human and mice proteins was generally >90%. PROK2-immunized SJL/J mice responded with predominantly IL-17^+^ T cells and developed a significant infiltration not only in the brain but also in the spinal cord. A similar trend has been demonstrated in MS (*7*, *59*). However, in EAE models in C57BL/6 and especially in C3H/HeJ, a higher T_H_17/T_H_1 ratio favors brain infiltration (*60*).

MHC is the most important genetic locus conferring MS susceptibility, specifically HLA-DRB1*15:01 and the tightly linked HLA-DRB5*01:01 being the major known risk factors while HLA-A*02:01 being the most substantial protective one (*34*). However, we could not detect any effect of these genotypes on the autoantigen responses in our experimental setup, meaning that while certain HLA genes increase or decrease the likelihood of developing an autoimmune response, it does not necessarily lead to a stronger or weaker response once it occurs. The use of full-length proteins for stimulation implies that intracellular processing and loading of peptides are optimal for each individual’s respective HLA, likely reducing the dependence of HLA–T cell epitope matching compared to stimulation with synthetic peptides. There is no singular HLA allele that is ubiquitous or completely protective in MS, meaning that epitopes driving disease are likely heterogeneous and can be presented by a large variety of HLA molecules. Nonetheless, the presentation and recognition of autoantigen epitopes were considerably HLA-DR restricted, which is in line with most HLA risk variants being DR alleles (*34*). Furthermore, full-length proteins in this context are an advantage over synthetic peptides, as overlapping peptide libraries are still limited in the number of possible peptides one can use. In addition, in silico prediction of HLA binding is not perfect. Hence, immunogenic peptides relevant in vivo might be overlooked using either peptide-based strategy. In addition, intracellular degradation of bead-bound full-length antigens increases the expression of MHC class II and costimulatory molecules of antigen-presenting cells, further enhancing antigen presentation and T cell activation over peptide stimulations (*61*).

Males had an overall stronger response to the autoantigens in our study. At a glance, this is seemingly at odds with the well-established observation that females are substantially more at risk of developing MS. However, studies have shown males to have a more aggressive disease course with a worse prognosis (*62*–*64*), fitting with the increased proinflammatory autoreactive T cells. In contrast, there were no strong correlations between other clinical characteristics and autoantigen responses. However, the considerable heterogeneity in the data requires a large cohort to allow for sufficiently powered analyses.

Comparison of FABP7 and SNAP91 reactivity in the untreated cohort did not achieve significance in one of the two comparisons, because of insufficient cohort size (Fig. 3A); however, clear trends were observed for increased reactivity to autoantigens in the patient group. The control groups did not differ in their responses, and increasing the number of donors in the control group by pooling HC and OND yielded even lower *P* values. Another example of this was the failure to detect significantly increased responses to the classical MS autoantigen PLP in one of the comparisons (*P* = 0.048 and *P* = 0.1096, compared to the pooled *P* = 0.035), indicating the insufficient power that the nonsignificant trends of the individual control groups were a result of.

While no single autoantigen was prominent in discriminating between MS and non-MS in this setup, a plurality of responses was able to clearly distinguish between the two, with an ROC test displaying an AUC of 0.86 to 0.90. This level of detection could be comparable to existing CSF biomarkers (*65*). However, confirmation is needed by comparing patient groups more relevant to a clinical setting (e.g., MS versus neuromyelitis optica), which was beyond the present study’s scope. Since completion of our screening experiments, further autoantigens have been found (*12*–*14*). While not included in the current study, these autoantigens should be included in future inquiries investigating the diagnostic potential of autoantigen reactivity in MS. Autoreactive profiles were personal, and no distinct patterns were observed, consistent with MS immunology being considered heterogeneous. The current paradigm proposes that there are probably several relevant immune targets and possibly no particular “silver bullet” antigen driving disease development, which our findings support. Even if there is a single triggering autoantigen, MS disease is likely to start silently years before clinical onset and, via mechanisms such as epitope spreading (*66*), may start an immunological cascade that leads to broader autoreactivity and subsequent clinical disease. It is possible that the recorded autoreactive responses are the result of such a spread, caused by an initial neuroinflammatory insult by autoreactive responses to any autoantigen to the CNS. This prompts further experiments elucidating this issue. However, capturing these early immunological events is practically impossible with present diagnostic tools but would be of key importance for both autoantigen discovery and early diagnostic profiling for personalized therapeutic approaches in MS. This broad autoreactivity also means that effective antigen-specific immunotherapy will likely require tolerization of several targets to be effective (*67*). This could pose a problem for immunotherapy as there are still likely knowledge gaps in the autoantigen repertoire. However, as MS likely hinges on the sum of several autoantigen responses, indicated by the several years long subclinical phase during which epitope spreading occurs, suppressing a part of them could be sufficient to reduce disease activity. In addition, there are options for the so-called bystander tolerization, which has been demonstrated in EAE (*68*). Still, the semi-high throughput of the autoantigen panel used in the present study could potentially be used as a pretreatment screening tool for designing personalized tolerization strategies.

In summary, the present study has contributed four previously unidentified proteins to the known MS autoantigen repertoire by demonstrating that FABP7, PROK2, RTN3, and SNAP91 are T cell targets in MS. No particular autoantigen proved to be a ubiquitous target, strengthening the hypothesis that MS is an immunologically heterogeneous disease and the result of multiple different avenues of T cell attack on the CNS. These novel autoantigens could pave the way for rapid and effective diagnostic tests and provide targets for future antigen-specific tolerization immunotherapies.

## METHODS

### Experimental design

The objective of this study was to identify novel T cell autoantigens in MS using an unbiased screening approach. We used a novel antigen-processing method to increase sensitivity and screened for T cell reactivity in vitro in pwMSs and HCs against a panel of 63 different CNS-expressed proteins. Hits from the screening were validated using full-length recombinant versions as antigens in two separate validation cohorts. Furthermore, the results were validated using a flow cytometry–based method, and autoreactive T cells were characterized. Experimental results were correlated with retrospectively collected clinical data. Last, the encephalitogenic potential of the found autoantigens was examined in two mouse models. Detailed description of data exclusions is available in later sections. There was no blinding in the main experiments, experiments on MS and control samples were always done in parallel to avoid potential experimental bias, and the settings for reading the results (e.g., FluoroSpot reader) were decided before the study and kept consistent throughout. Immunohistochemistry analysis of mouse samples was blinded in regard to which immunization or control immunization each group received.

### Human subjects

The screening cohort consisted of natalizumab-treated pwMSs (*n* = 16) and age- and sex-matched HCs (*n* = 9). The first validation cohort consisted of natalizumab-treated pwMSs (*n* = 61) and age- and sex-matched HCs (*n* = 28). The second validation cohort consisted of untreated pwMSs (*n* = 30), persons with OND (*n* = 19) undergoing investigation for sleep disorders (retrospective control of diagnosis in this group resulted in *n* = 10 narcolepsy type 1, *n* = 3 narcolepsy type 2, and *n* = 6 hypersomnia with unclear diagnosis), and a representative sampling of HCs from the first validation cohort (*n* = 20). The demographics of these cohorts are presented in table S2. All included pwMSs had diagnosed relapsing-remitting MS according to the McDonald 2010 criteria. Patients and controls were recruited at the Karolinska University Hospital, Stockholm, Sweden, and Academic Specialist Center, Stockholm, Sweden. A part of the untreated MS cohort was recruited at the University Hospital in Zürich, Switzerland. Clinical data were obtained retrospectively from medical records, blinded in regard to experimental results. HLA status was obtained from existing records of previous studies. HLA data remained unavailable for some individuals (table S2). Clinical data were unavailable for one pwMSs in the natalizumab-treated cohort and one in the untreated cohort. The sample collection was approved by the regional ethics board in Stockholm (nos. 2009/2107-3112 and 2015/1161-31/4) and the Cantonal Ethics Committee of Zürich (no. 2013-0001). Sera for the antibody analysis were collected separately and approved by the regional ethics board in Stockholm (no. 04-252/1-4).

### Cell preparation

PBMCs were isolated from EDTA-containing blood sampling tubes within 2 hours after sampling or leukapheresis (untreated MS, *n* = 6) via Ficoll density gradient centrifugation as previously described (*29*). All cells were cryopreserved in freezing medium containing 10% dimethyl sulfoxide, 45 or 90% fetal bovine serum, and 0 or 45% RPMI 1640 (Sigma-Aldrich), frozen at a rate of −1°C/min, and stored at −150° to −180°C.

### Autoantigen screening panel

The antigens for initial screening consisted of PrESTs from the HPA project (*31*). Briefly, the PrESTs consisted of protein fragments chosen for low sequence similarity with other proteins as described (*32*) and contained an albumin-binding protein and hexahistidine tag for purification (His6-ABP-PrEST). PrESTs were selected by four criteria: (i) established MS autoantigens, (ii) previously studied candidates, (iii) selectively CNS-expressed proteins based on the HPA data (www.proteinatlas.org, obtained in the first quarter of 2013), and (iv) proteins of interest based on communication with experts in the field. The final screening panel consisted of 124 PrESTs from 63 human proteins, which were grouped into 45 pools; the full list of included PrESTs is presented in table S1. Before pooling, the PrESTs were coupled to 1-μm paramagnetic beads (Dynabeads MyOne, Thermo Fisher Scientific) as previously described (*30*) and washed five times each with solutions containing 2 M sodium hydroxide (NaOH), 1% Triton X-100, 0.5 M l-arginine, and, lastly, sterile phosphate-buffered saline (PBS) to remove bacterial contaminants.

### Design of the full-length autoantigen panel

The control antigens NC (*69*) and CMV (*70*) were designed as previously described (*30*). The autoantigens were designed as corresponding genes, including flanking Bsa I sites, omitting eventual signal peptides. In the case of PROK2, two splicing variants were included. For RTN3 and SNAP91, the proteins were divided into two and four different parts, respectively, for production purposes. For PLP, transmembrane regions were omitted from the design. The genes were subcloned into an albumin-binding domain (ABD)-containing modified pET28 vector (Merck Millipore) as in (*71*), resulting in a fusion protein containing a hexahistidine tag, ABD, and antigen, all interspaced with glycine-serine linkers (His6-ABD-protein). The full design of the panel is available in table S3.

### Antigen production and purification

The vectors were transformed into BL21-AI *Escherichia coli* (Thermo Fisher Scientific) and grown in supplemented super broth medium overnight at 25°C with autoinduction as previously described (*29*). Cells were lysed using a lysis buffer and a freeze-thaw cycle, followed by subsequent purification of the protein using His Mag Sepharose Ni beads (Cytivia, formerly GE Healthcare). After elution, the protein eluates were immediately adjusted to pH 5 by the addition of an MES buffer and analyzed for purity by SDS–polyacrylamide gel electrophoresis (fig. S1A) and concentration by NanoDrop (Thermo Fisher Scientific).

### Antigen preparation

The antigens were coupled to 1-μm paramagnetic polystyrene beads with carboxyl acid groups on the surface (Dynabeads MyOne, Thermo Fisher Scientific) via amine coupling as previously described (*30*). Briefly, the carboxylic acid was preactivated by incubation with *N*-hydroxysuccinimide (NHS) and 1-ethyl-3-(3-dimethylaminopropyl)carbodiimide (EDC) before the addition of the antigen at a 1:15 weight ratio of antigen to beads. After 30 min of incubation at room temperature, remaining carboxylic acid groups were quenched by the addition of tris buffer. After coupling, the antigen beads were washed four times in a NaOH solution to remove endotoxin contaminants. The NaOH concentration and an additional wash with 1% Triton X-100 were individually determined for each antigen via testing previously reactive patients with MS from the screening cohort and HCs for optimal signal-to-noise ratios. Final wash conditions for the autoantigen panel are available in table S3. After endotoxin removal, the beads were washed five times in sterile PBS. The antigen coupling was determined using a bicinchoninic acid (BCA) test (catalog no. 23235, Thermo Fisher Scientific), with tris- and bicine-deactivated beads as a control. The BCA test was performed according to the manufacturer’s instructions in microtubes, at 55°C for 50 min on a shaker at 1500 rpm before removing the beads and transferring the supernatant to a 384-well plate. All tests were performed in triplicates. Absorbance at 562 nm was read using a SpectraMax Plus 384 (Molecular Devices) (fig. S1B). The remaining LPS contamination was measured using a Limulus amebocyte lysate assay (Endpoint Endochrome Kit, R160K, Charles River Laboratories) (fig. S1C). The assay was performed according to the manufacturer’s instructions, with the addition of a 95°C incubation step for 5 min for the beads before testing to release as much LPS as possible. Both the beads and supernatant were tested together for maximum detection.

### FluoroSpot assays

Cryopreserved PBMCs were briefly thawed in a water bath at 37°C before washing twice in RPMI 1640 medium (R8758, Sigma-Aldrich) supplemented with 10% heat-inactivated fetal bovine serum (F7524, Sigma-Aldrich), 2 mM l-glutamine (G7513, Sigma-Aldrich), and penicillin (100 U/ml) and streptomycin (100 μg/ml) (P4333, Sigma-Aldrich). Cell count and viability were measured by trypan blue staining using an automated counter (LUNA-II, Logos Biosystems). Median viability was 88.5 to 90% and did not differ between the cohort groups (fig. S1D). A total of 250,000 viable cells in 100 μl of cRPMI per well were added to a precoated and blocked IFNγ/IL-22/IL-17A FluoroSpot plate (FSP-011803, Mabtech) containing 100 μl of cRPMI and 3 × 10^6^ antigen beads. In cases where more than one type of antigen bead was used, the types were distributed equally with a total of 3 × 10^6^ beads. For the anti-CD3 positive control, 125,000 PBMCs were added to each well. The plates were incubated for 44 hours at 37°C and 5% CO_2_. Each test was performed in duplicates, and MS samples were always tested in parallel with control samples on the same plate. After incubation, the plates were developed according to the manufacturer’s instructions. The FluoroSpot plates from the screening and the first validation were read using an iSPOT Spectrum device (Advanced Imaging Devices GmbH, Germany) using the AID EliSpot Reader v.7 software (Advanced Imaging Devices GmbH), while the rest were read using an IRIS plate reader (Mabtech, Sweden) and the SpotReader v.1.1.9 software (Mabtech). Each individual’s response to the NC beads was subtracted from the spot count in the antigen-stimulated wells and is presented as delta SFUs (ΔSFUs).

The HLA blocking was performed similarly in the FluoroSpot assay, with 10 μg/ml of anti–HLA-DR (BioLegend) or anti–HLA-A/B/C (BioLegend) antibodies during incubation. As an NC for the blocking, a mouse IgG2a isotype control was used (BioLegend). Both natalizumab-treated (*n* = 12) and untreated pwMSs (*n* = 3) were tested. Tests were excluded from analysis if unresponsive in the autoantigen + isotype control (<6 ΔSFUs for IFNγ and <4 ΔSFUs for IL-17A).

### Flow cytometry

Cryopreserved PBMCs were thawed, washed, and counted as previously described before being seeded in a 96-well V-bottom plate at 2 × 10^5^ cells per well. Anti-CD3 antibody (0.1 μg/ml; mAbCD3-2), autoantigen beads (10:1 bead:cell ratio), or no stimulus was added to appropriate wells before incubation for 44 hours at 37°C and 5% CO_2_. The concentration of stimulatory mAbCD3-2 was kept low so as not to interfere with subsequent fluorescent CD3^+^ staining. Brefeldin A (420601, BioLegend) was added to all wells at 1 μg/ml for the final 16 hours of incubation. Cells were first stained with live/dead fixable aqua stain (L34957, Life Technologies) (diluted 1:1000 in PBS) before staining for surface markers [CD3-APC-Cy7, CD4-PerCP-Cy5.5, and CD8–phycoerythrin (PE)–Cy7, BioLegend]. After fixation using intracellular fix buffer (420801, BioLegend), cells were permeabilized using perm wash buffer (421002, BioLegend) before intracellular cytokine staining [IFNγ–fluorescein isothiocyanate (FITC), IL-17A–BV421, IL-22–PE, and GMCSF–APC, BioLegend]. Last, samples were washed and acquired on a BD FACSVerse flow cytometer (BD Biosciences, USA) using the BD FACSuite v.1.0.6 software (BD Biosciences). The gating strategy is presented in Fig. 4A. All staining incubations were performed for 30 min at 4° (before fixation) or at room temperature (after fixation). All centrifugation wash steps were performed at 300*g* for 5 min (before fixation) or 500*g* (after fixation). All raw flow cytometry data were analyzed using FlowJo version 10 software (BD Biosciences).

### Autoantibody suspension bead array

A suspension bead array assay was used to examine plasma from a large cohort consisting of pwMSs (*n* = 518) and HCs (total *n* = 554) for potential autoantibodies targeting the novel autoantigens. The antigens used in the FluoroSpot assay (table S3) were included, omitting the PROK2 isoform 2, as the whole sequence is contained in the PROK2 isoform 1, as well as PLP, because of solubility issues. In addition, PrESTs of the same proteins from the HPA were included. As NCs, the 6xhistidine-ABD tag (NC tag) and buffer were only used, while rabbit anti-human IgG (309-005-082, Jackson ImmunoResearch) served as a positive loading control. Eighteen proteins belonging to a separate, unrelated experiment were analyzed in the same combined assay and were included in the calculation of background (see below). All antigens were coupled using NHS-EDC coupling to color-coded magnetic beads (MagPlex, Luminex Corp.), with unique IDs for each antigen. Coupling efficiency was evaluated by measuring the ABD tag present on all PrESTs and proteins. If negative, then it was further tested using protein-specific antibodies from the HPA. All antigens were confirmed with at least one method, except SNAP91_750–904_ for which no antigen-specific antibody was available.

Plasma samples and commercial plasma (mixed 1:1 male and female; PK2-123-V-37973, Seralab) as a technical control were diluted 1:150 in buffer [PBS supplemented with 5% bovine serum albumin (BSA), 0.1% Tween 20, NC tag (160 μg/ml), and neutravidin (10 μg/ml)] and incubated for 1 hour at room temperature for the soluble NC tag and neutravidin to preblock any potential antibodies. The sera were incubated with the antigen bead array for 2 hours, and antibody binding was subsequently fixed using 0.2% paraformaldehyde for 10 min. An R-PE–labeled secondary reagent (goat anti-human IgG Fab fragments labeled with R-PE, H10104, Invitrogen) was applied for 30 min, and the beads were analyzed using a FlexMap 3D (Luminex Corp., Austin, TX). The readout consisted of the median fluorescent intensity (MFI) of each individual bead ID. The commercial plasma was included in triplicates in each sample plate. The coefficient of variation across the antigens was 4.6 to 9.6%.

Before further analysis, to compensate for differences in background signal, the raw MFI data were transformed using the assumption that in any given plasma sample, one-third of antigens would not be reactive. Hence, raw MFI values were transformed per individual into Per33 MFI, calculated as MFI of the sample minus the 33rd percentile MFI of all samples (*n* = 43). To calculate a proportion of positive autoantibody responses, a cutoff for positivity was set at the means + 3 SD of the buffer, resulting in a cutoff of ≥2350 MFIs.

### Enzyme-linked immunosorbent assay

To validate the high antibody titers to RTN_1–300_, an independent enzyme-linked immunosorbent assay was performed. As antigen, a recombinant version of RTN_1–300_ omitting the ABD tag was used. MaxiSorp plates were coated overnight with 100 μl of RTN3_1–300_ or BSA (5 μg/ml; no. SH30574.02, HyClone), washed five times, and blocked with 1% BSA for 1 hour. Plates were washed five times before sera from patients with MS (*n* = 11) and HCs (*n* = 11), diluted 1:30 in PBS containing 0.2% BSA and 0.05% Tween 20, and were added and incubated for 2 hours at room temperature. Plates were again washed five times before developing using horseradish peroxidase–conjugated goat anti-human IgG (catalog no. 109-035-003, Jackson ImmunoResearch) for 1 hour and secondary 3,3′,5,5′-tetramethylbenzidine (Sigma-Aldrich) for 10 min before stopping the reaction with 100 μl of 0.5 M H_2_SO_4_. Plates were read at 450 nm using a SpectraMax Plus 384 (Molecular Devices, USA) using the SoftMax Pro 7.03 software (Molecular Devices). All tests were performed in duplicate. Each individual’s optical density in the BSA wells was subtracted from the RTN_1–300_ wells before statistical analysis.

### In silico basic local alignment search tool

To identify possible cross-reactivity between RTN3_1–300_ and pathogens, a protein-protein basic local alignment search was performed using the open-source software BLASTP 2.11.0+ (National Center for Biotechnology Information, https://blast.ncbi.nlm.nih.gov) (*72*). The RTN3 sequences 1 to 90 and 207 to 266 were used as queries in the search. The search was performed using the nonredundant protein sequences database, with an expected threshold of 0.05, a word size of 2, and the BLOSUM62 matrix. Results were filtered to include bacteria and viruses.

### Animals, immunizations, and EAE assessment

SJL/J and DBA/1 female mice were purchased from Taconic Biosciences. All animals were kept in 12-hour light/dark- and temperature-regulated rooms, housed in polystyrene cages containing aspen wood shavings and with access to standard rodent chow and water ad libitum.

For the induction and clinical evaluation of EAE, animals were immunized subcutaneously at the tail base with antigen pools in 100 μl of Freund’s complete adjuvant (FCA, Chondrex) containing 200 μg of heat-killed *Mycobacterium tuberculosis* (MTB) per mouse as follows: FABP7 and RTN3 at 100 μg per animal; PROK2 and SNAP91 at 70 μg per animal; NC tag at 70 μg per animal as a control for PROK2 and SNAP91. For PROK2, RTN3, and SNAP91, mixes of the different isoforms or protein parts were used, with the total protein amount as mentioned above. For FABP7 and RTN3, versions without the NC tag were used. Homologies between the human versions used and the corresponding mouse sequences were generally high: 94.4% homology for PROK2, 94.7% for SNAP91, 80.9% for FABP7 isoform 2 (93.9% for isoform 1), and 74.3% for RTN3. All animals received two injections of pertussis toxin (Calbiochem) at 200 ng per animal in 200 μl of PBS intraperitoneally at day 0 and day 2 after immunization. As positive controls for the induction of EAE and immunofluorescence, DBA/1 animals were immunized with recombinant mouse MOG_1–125_ at 20 μg per animal in FCA containing 50 μg of heat-killed MTB per animal without pertussis toxin administration, and SJL/J animals were immunized with PLP_139–151_ at 50 μg per animal in FCA containing 200 μg MTB per animal without pertussis toxin administration. Three to eight animals were used per antigen. Animals immunized with control antigens were scored and weighed daily after disease onset, while animals immunized with the novel MS candidate antigens were observed three times weekly from day 10 after immunization for any abnormalities in tail tonus and gait. The clinical score was graded as follows: 0, no clinical signs of EAE; 1, tail weakness or tail paralysis; 2, hind leg paraparesis or hemiparesis; 3, hind leg paralysis or hemiparalysis; 4, tetraplegia or moribund; 5, death. All experiments were approved by the Swedish National Board for Laboratory Animals (N138/14) and performed in accordance with the European Community Council Directive (86/609/EEC).

### Assessment of antigen-specific responses in mice

On day 40 after immunization, animals were sedated with isoflurane, perfused transcardially with 50 ml of PBS, and the brain, spinal cord, and spleens were dissected for further experiments. Splenocytes were mechanically dissociated, and cells were spun and resuspended in RPMI 1640 complemented with 5% fetal bovine serum, 1% l-glutamine, 1% penicillin-streptomycin, 1% pyruvic acid (all from Sigma-Aldrich), and 50 mM 2-mercaptoethanol (Gibco-BRL). Cells were plated at 1 × 10^6^ cells per well in U-bottom plates and restimulated with the immunization antigen and a control antigen, NC (as a control for PROK2 and SNAP91), or no antigen. FABP7 and RTN3 were added as recombinant proteins (20 μg/ml), while PROK2, SNAP91, and the NC protein control were added as bead-coupled antigens (12 × 10^6^ beads per well). After 96 hours, cells were replated into V-bottom plates and restimulated for 5 hours with phorbol 12-myristate 13-acetate (50 ng/ml; Sigma-Aldrich), ionomycin (1 μg/ml; Sigma-Aldrich), and brefeldin A (GolgiPlug) (1 μl/ml; BD Biosciences). Samples were subsequently stained with the following conjugated antibodies: CD3-APC-Cy7, CD4-PerCP-Cy5.5, FoxP3-PE-Cy7, Ki67-V450 (all from BD Biosciences), GM-CSF–FITC, IL-17–PE, and IFNγ-APC (all from eBioscience). LIVE/DEAD Fixable Near-IR Dead Cell Stain (L34976, Invitrogen) was used to exclude dead cells. Intracellular/intranuclear staining was performed after permeabilization using a fixation/permeabilization kit (BD Biosciences/eBioscience). Cells were acquired using a Gallios flow cytometer (Beckman Coulter) and analyzed using Kaluza software (Beckman Coulter).

### Immunofluorescence

Paraffin-embedded, 5-μm-thick brain and spinal cord cross sections were dewaxed in xylol, rehydrated, and subsequently stained with hematoxylin and eosin to assess inflammatory infiltration. Immunotargeting of CD45 was performed in adjacent tissue sections using an antibody against CD45 (rabbit anti-CD45, ab208022, Abcam) as previously described (*73*). Briefly, after deparaffinization in xylol, sections were rehydrated to distilled water via ethanol with increasing water content.

Antigen retrieval in citrate buffer (pH 6.0) was performed in a steamer device (Braun, Germany) for 45 min. Sections were incubated in 10% fetal bovine serum in 0.1 M PBS 30 min before incubation with primary antibody at 4°C overnight. Control sections were incubated in the absence of the primary antibody. After washing in PBS, sections were incubated with secondary antibody (donkey anti-rabbit, Invitrogen) conjugated with Alexa Fluor 488. DAPI (4′,6-diamidino-2-phenylindole, dihydrochloride; 0.2 μg/ml) was included in the last PBS washing step to visualize the nuclei. Stained tissue sections were mounted using ProLong Gold Antifade reagent (Molecular Probes). All images were acquired using a Leica SP5 confocal microscope with the software Leica application suite X (Leica). The quantification of the immune cell infiltration in mice treated with different antigens represents the number of CD45^+^ cells per square millimeter counted on an average of five to six brain or whole–spinal cord cross sections per animal. The image analysis of the location of infiltrating leukocytes was performed similarly, including staining blood vessels using goat anti-podocalyxin red (R&D Systems).

### Statistical analysis

Statistical analyses were performed using Prism 7 and 9 (Figs. 2, 3, and 6) (GraphPad, USA) software. The significance threshold was set as *P* < 0.05 for all tests. If not stated otherwise, then non-normal distribution was assumed, and two-tailed tests were applied. For identifying candidates in the screening, two-way analysis of variance (ANOVA) with Sidak correction was used, and antigens with at least one cytokine with *P* < 0.05 were chosen for the full-length panel. For the validation experiments, nonparametric two-tailed Mann-Whitney *U* tests were used, with correction for multiple comparisons using false discovery rate (FDR) (Benjamini and Hochberg) with a desired FDR = 5%. The adjusted *P* values (FDR *q* values) are reported. The composite ROC test was made by using the ROC analyses of single autoantigens to determine optimal ΔSFU cutoffs via a convex hull approach (*74*). For other analyses, nonparametric two-tailed tests were also used, i.e., Mann-Whitney *U* test to compare cohorts, Wilcoxon signed-rank test for dependent values, and Spearman test for correlations. When calculating the ratio between CD4^+^/CD8^+^ T cells, in cases where no IFNγ^+^ CD8^+^ T cells were detected, the lowest detected autoantigen response for that individual was used as a placeholder. For antigen recall experiments in the mouse model, a one-way ANOVA with Tukey’s correction for multiple comparisons was used, comparing each response with all others. For determining leukocyte infiltration, one-way Brown-Forsythe and Welch ANOVA with Holm-Sidak correction for multiple comparisons was applied because of the differences in group sizes and SDs, comparing autoantigen-immunized groups to the NC-immunized mice. Detailed information and results of all main statistical tests are available in data files S1 to S7.

### Detailed description of data exclusions

For FluoroSpots, tests with >30 detected spots for any cytokine in the NC (NC beads) or <150 IFNγ spots in the positive control were retested; if still out of acceptable range, then they were excluded from analysis (two pwMSs in the screening cohort, two pwMSs and one HC from the first validation, and two pwMSs, one HC, and one OND from the second validation). Samples not responding to the polyclonal stimulation were assumed to be of too poor quality and resulting in false negatives. If they have very high background responses, then it was likely due to contamination of the samples, giving general activation and false high responses.

All FluoroSpot plate images were manually inspected to remove potential artifacts (dust, cloth fibers, etc.) that are otherwise counted as spots by the software. A few complete wells were manually excluded after reading the FluoroSpot plate because of unknown widespread artifacts that generated border-adjacent confluent spots and overall high fluorescence in the well (two untreated patients with MS and one with OND, four antigen stimulations in two, and three antigen stimulations in another; visible in Fig. 3E and raw data files). This was all done to reduce false positives as the number of spots automatically counted was high. In HLA-blocking experiments, samples with very low responses in the isotype control + antigen were excluded from further analysis, as it was assumed to be background rather than antigen-specific responses.

The suspension bead array experiment was run in four separate assays. Commercial plasma was always included to verify comparable responses. One of the four assays had lower signals and increased variability, especially when looking at the positive general human IgG control bead and was therefore excluded. Patients and controls were randomized, and exclusion of one of the assays should not bias the results.

In the flow cytometry experiments, the MBP-bead stimulation condition was excluded as no response was seen in any individual. As this was in stark contrast to the other stimulations and the FluoroSpot data, it was considered an outlier because of a technical error and was excluded from further analysis.
