# Supplementary material for: Identification of four novel T cell autoantigens and personal autoreactive profiles in multiple sclerosis
Source: Sci Adv. 2022 Apr 27;8(17):eabn1823. doi: 10.1126/sciadv.abn1823 (PMC9045615; doi:10.1126/sciadv.abn1823)
Supplement: Supplementary file 1 — Figs. S1 to S7 Tables S1 to S3 [file sciadv.abn1823_sm.pdf]

Science Advances
AAAS

Supplementary Materials for

Identification of four novel T cell autoantigens and personal autoreactive profiles in multiple sclerosis

Mattias Bronge*, Klara Asplund Högelin, Olivia G. Thomas, Sabrina Ruhrmann, Claudia Carvalho-Queiroz, Ola B. Nilsson, Andreas Kaiser, Manuel Zeitelhofer, Erik Holmgren, Mathias Linnerbauer, Milena Z. Adzemovic, Cecilia Hellström, Ivan Jelcic, Hao Liu, Peter Nilsson, Jan Hillert, Lou Brundin, Katharina Fink, Ingrid Kockum, Katarina Tengvall, Roland Martin, Hanna Tegel, Torbjörn Gräslund, Faiez Al Nimer, André Ortlieb Guerreiro-Cacais, Mohsen Khademi, Guro Gafvelin, Tomas Olsson, Hans Grönlund

*Corresponding author. Email: mattias.bronge@ki.se

Published 27 April 2022, *Sci. Adv.* **8**, eabn1823 (2022)
DOI: 10.1126/sciadv.abn1823

The PDF file includes:

Figs. S1 to S7
Tables S1 to S3
Legends for data S1 to S8

Other Supplementary Material for this manuscript includes the following:

Data S1 to S8

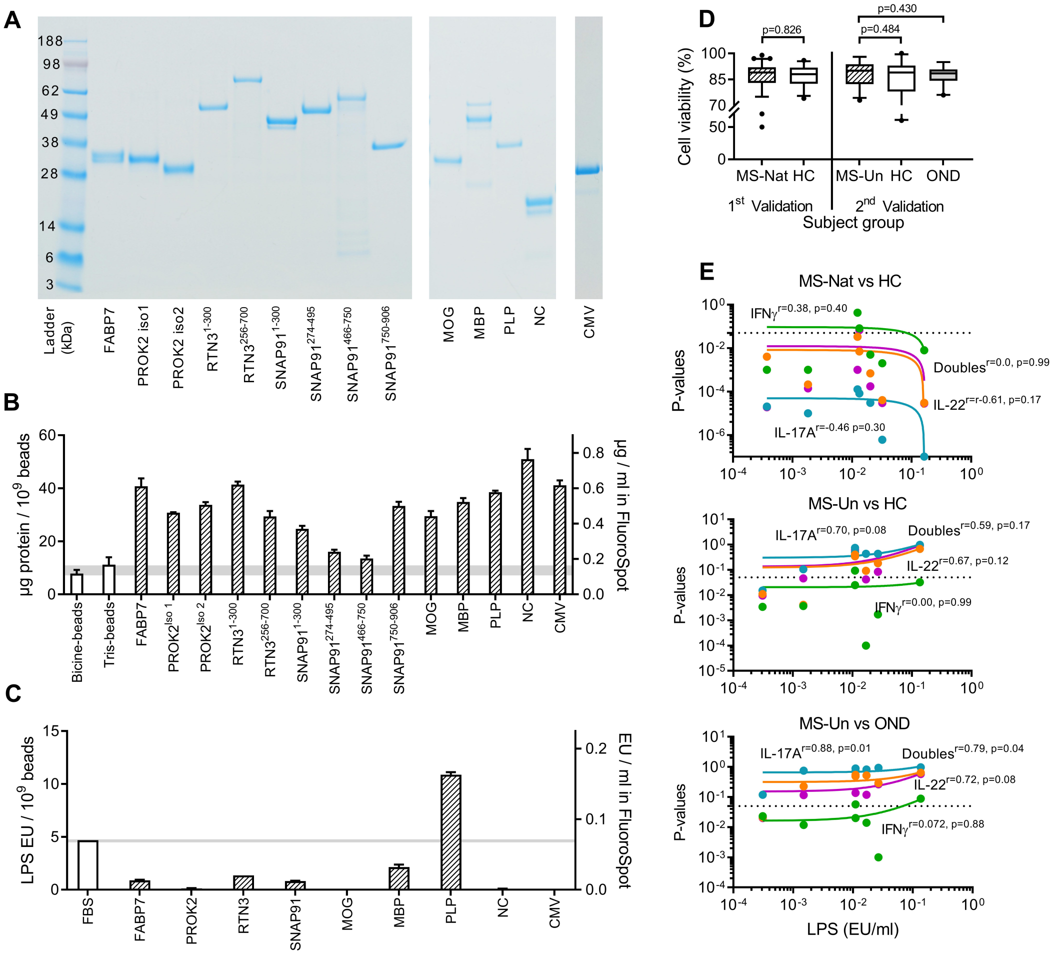

Fig. S1. Full-length antigen panel quality control. **A**, SDS-page gels of the full-length antigens used in the FluoroSpot-assay validations. Molecular size ladder with size indicators in the leftmost lane. Picture derived from three different gels, each containing a lane with the same ladder (cropped out) and the bands' locations in relation to the ladder are consistent. Brightness, contrast, and colour temperature corrections have been made. **B**, The protein content of the final, endotoxin-washed antigen beads compared to beads coupled with bicine and tris. Bars represent the mean and staples the SD of the intra-assay replicates. Bars are plotted on both the left and right y-axis (resulting protein concentration in the FluoroSpot-assay). The horizontal gray line represents the negative control results. **C**, LPS concentration of the finished, endotoxin-washed, and pooled antigen-beads and the fetal bovine serum (FBS) used in the cell media. Bars represent mean and staples SD of the intra-assay replicates. LPS content in antigen-bead preparations is plotted on both the left (endotoxin units (EU) per 10^9 beads) and the right y-axis (resulting EU per ml in the FluoroSpot assay). FBS is only plotted on the right y-axis. The horizontal gray line represents the LPS (EU/ml) of the FBS. **D**, Comparison of the different cohorts' PBMC viability after thawing. Box represents median and IQR and brackets the 5-95 percentile. P-values calculated using a two-tailed Mann-Whitney U-test. **E**, Correlation of the p-values of the difference in autoantigen responses for the 1st (top graph) and 2nd (bottom graphs) validation runs and autoantigen LPS content. IFNγ in green, IL-22 in orange, IL-17A in blue, and double-cytokines in magenta. Lines represent linear regression slopes. Dotted line at p=0.05. P- and r-values were calculated using two-tailed non-parametric Spearman-correlation.

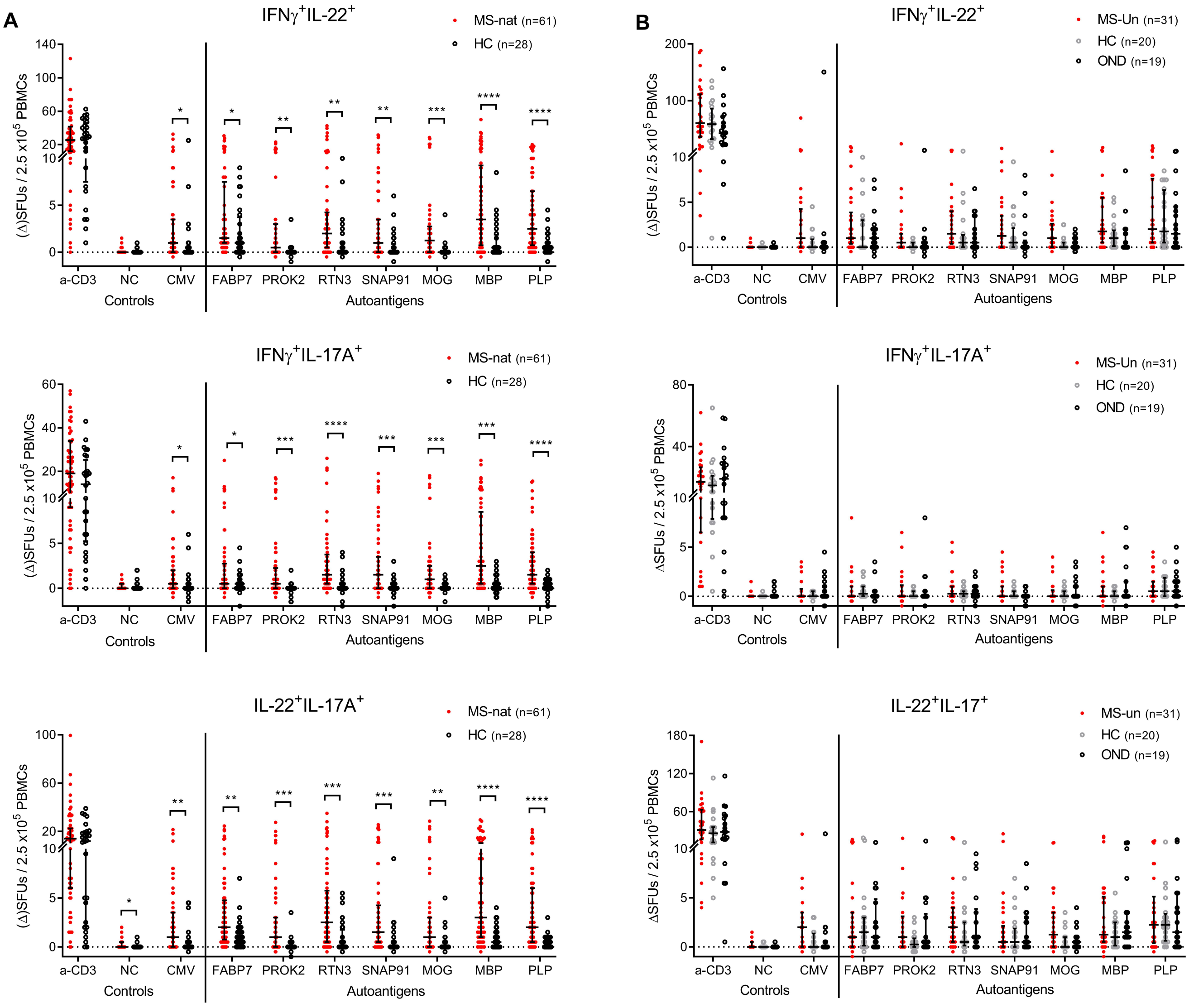

Fig. S2. Double cytokine-producing cells. Double cytokine-producing cells in the IFNγ/IL-22/IL-17A FluoroSpot assay. **A**, Results from the 1st validation cohort, using natalizumab-treated persons with MS. Each dot represents one individual with brackets representing median and IQR. The results from the anti-CD3 positive control stimulation are plotted as ΔSFUs / 1.25 x10^5 PBMCs and NC are plotted as raw SFUs / 2.5 x10^5 PBMCs. P-values were calculated with a two-tailed Mann-Whitney U-test with Bonferroni correction for multiple comparisons (n=10). *p<0.05, **p<0.01, ***p<0.001. **B**, Results from the 2nd validation cohort, using untreated persons with MS, presented as in (**A**).

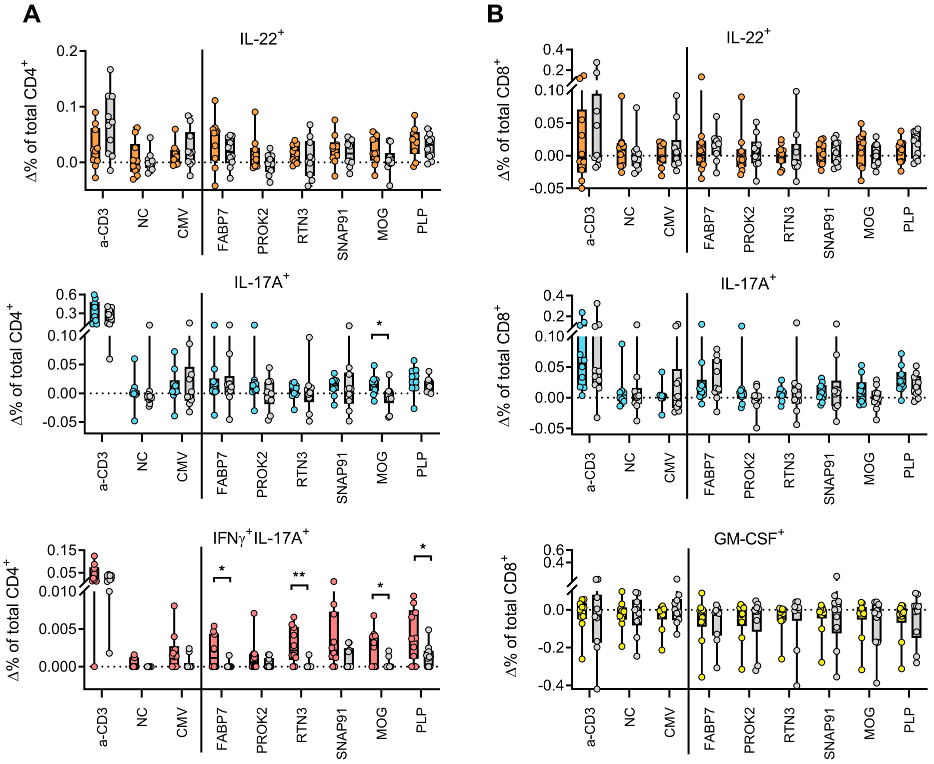

Fig. S3. Intracellular cytokine staining after autoantigen stimulation. Flow cytometry analysis of autoreactive T cells using intracellular staining after autoantigen stimulation. **A**, IL-22, IL-17A, and double-positive IFNγ$^+$IL-17A$^+$ responses in CD4$^+$ T cells after stimulation with autoantigens or control antigens. **B**, IL-22, IL-17A, and GM-CSF responses in CD8$^+$ T cells. Plotted as % of the total population after subtracting background responses (no stimuli, Δ%). Boxes represent median and IQR. MS (n=10) in color, HC (n=10) in gray open circles. P-values calculated using a two-tailed Mann-Whitney U-test and written when significant. *p<0.05, **p<0.01.

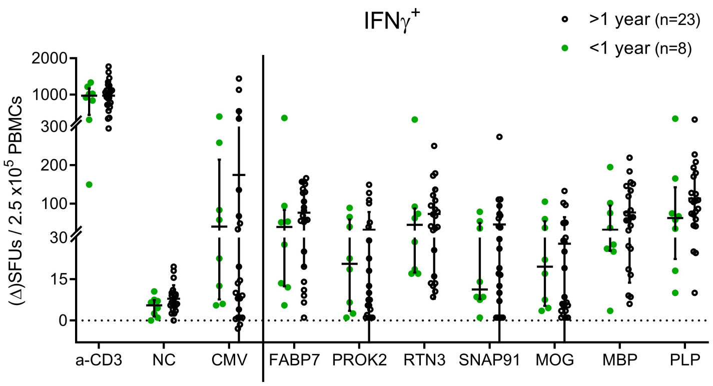

Fig. S4. Short versus long disease duration. IFNγ responses of untreated MS persons, stratified based on first known symptoms within one year of sampling (<1 year, mean and SD 0.49 ±0.18 years) or longer disease duration (>1 year, mean and SD 4.9 ±3.3 years). P-values were calculated using two-tailed Mann-Whitney U-tests and none were significant (p=0.22-0.94 in all cases).

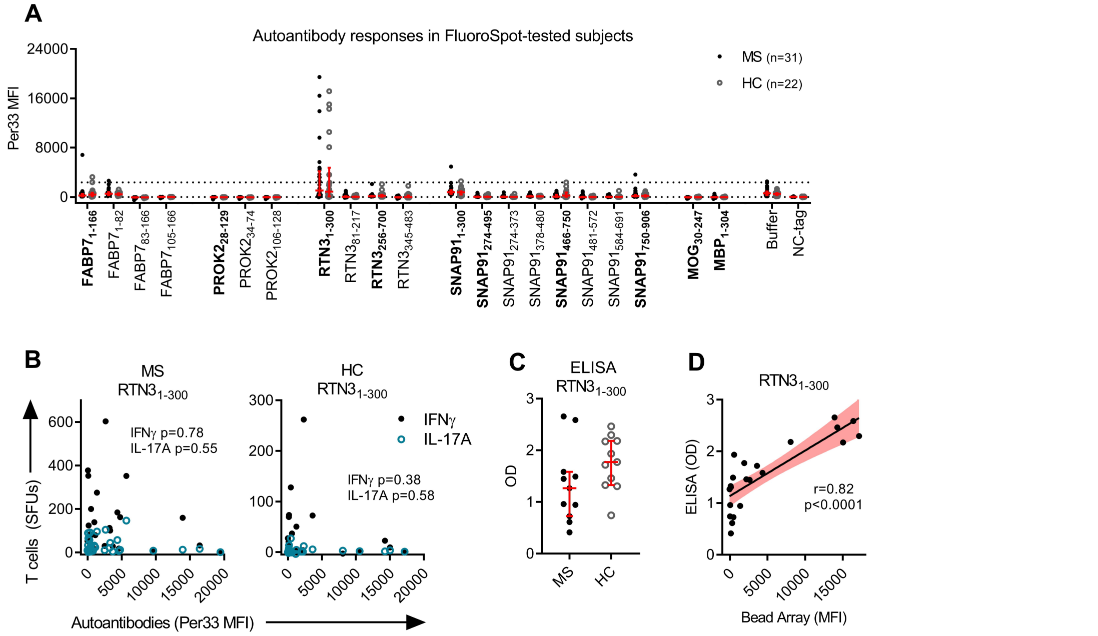

Fig. S5. Autoantibody and T cell correlation, and RTN3 validation. A, Results from the suspension bead array autoantibody detection in pwMS (n=31) and HC (n=22) which had also been tested for T cell reactivity using the FluoroSpot assay in the 1st validation cohort. Both full length antigens used in the FluoroSpot assay (x-axis, bold) and PrESTs (x-axis, not bold) were tested. The number denotes the amino acids covered in the protein. Dotted line at the threshold for positivity (MFI=2350). Brackets denote median and IQR. **B**, Correlation analysis between the suspension bead array (**A**) and FluoroSpot IFNγ (black filled circles) and IL-17A (blue open circles) responses to $RTN3_{1\text{-}300}$ for pwMS (left graph) and HCs (right graph). P-values calculated using a two-tailed non-parametric Spearman correlation. **C**, Enzyme-linked immunosorbent assay (ELISA) detecting autoantibodies to a second version of $RTN3_{1\text{-}300}$ which did not contain the NC-tag contained in the previous $RTN3_{1\text{-}300}$ version, testing both pwMS and HC (n=11 in each group) also tested in (**A**). **D**, Correlation of the results from ELISA (OD, y-axis) and values were calculated using a two-tailed non-parametric Spearman correlation. MFIs: Median fluorescent intensity.

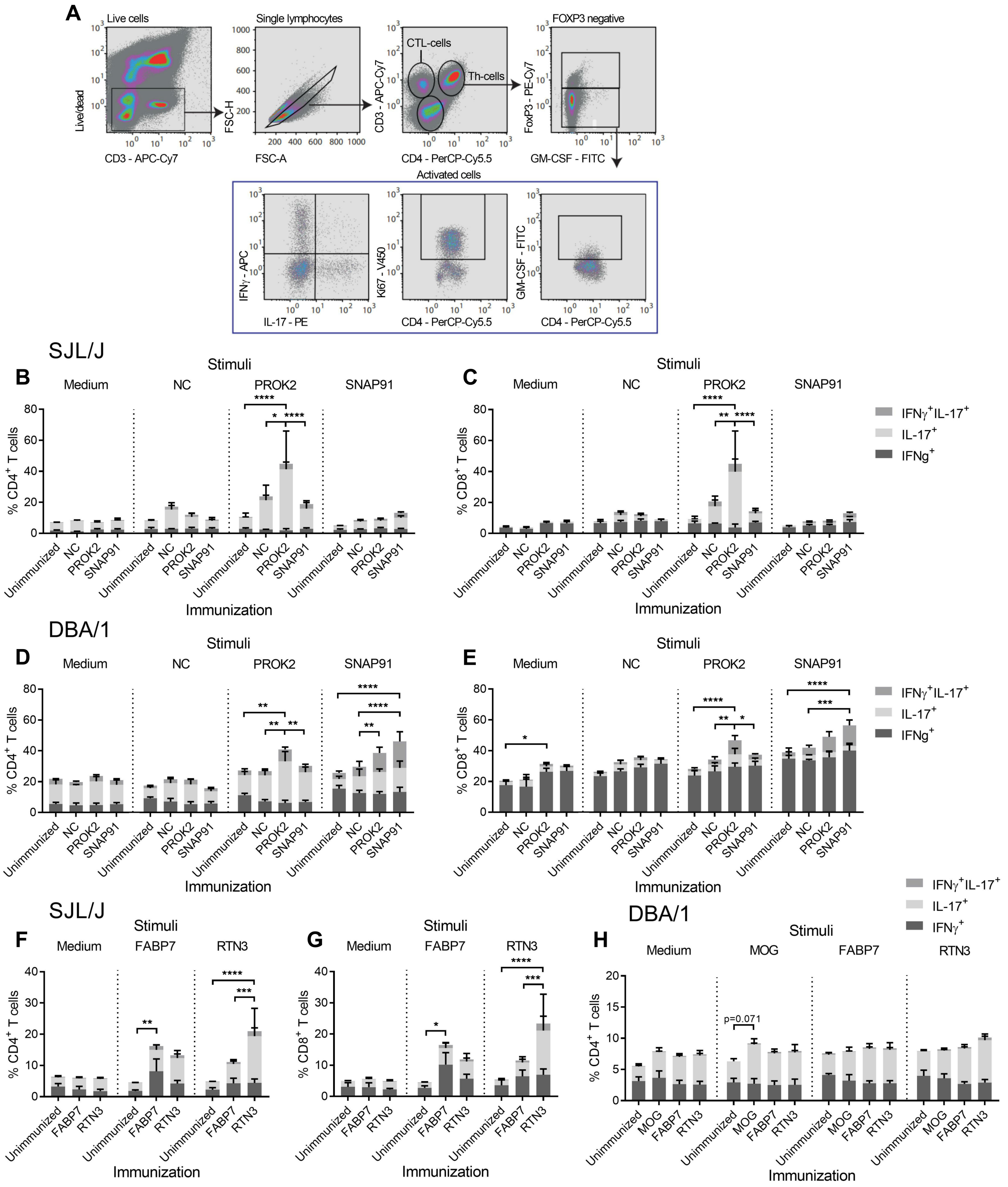

Fig. S6. Immune responses in autoantigen-immunized mice. Recall responses to the novel autoantigens in immunized mice. **A**, Gating strategy of cytokine responses. **B** and **C**, $CD4^+$ and $CD8^+$ T cell responses to medium only, NC, PROK2 and SNAP91 in unimmunized (n=3), NC (n=3), PROK2 (n=6) and SNAP91 (n=6) immunized SJL/J mice. **D** and **E,** Responses to medium only, NC, PROK2 and SNAP91 in unimmunized (n=3), NC- (n=3), PROK2- (n=6) and SNAP91- (n=6) immunized DBA/1 mice. **F** and **G**, Responses to medium only, FABP7 and RTN3 in unimmunized (n=3), FABP7 (n=7), and RTN3 (n=7) immunized SJL/J mice. NC was omitted as versions of FABP7 and RTN3 without the NC-tag were used for both immunisation and stimuli. **H**, $CD4^+$ responses to medium only, MOG, FABP7 and RTN3 in unimmunized

(n=3), MOG (n=3), FABP7 (n=8) and RTN3 (n=8) immunized DBA/1 mice. Stacked bar graphs represent the mean ±SD cytokine positive cells as % of the total $CD4^+$ or $CD8^+$ cell population. The different cytokine responses are stacked on top of each other. X-axes denote the immunisation and dotted lines separate the different stimuli used ex vivo. P-values calculated using a two-tailed ANOVA with Tukey's correction for multiple comparisons and written when significant. ANOVA test based on the total % of cytokine-producing cells, not individual cytokines. *$p<0.05$, **$p<0.01$, ***$p<0.001$, ****$p<0.0001$.

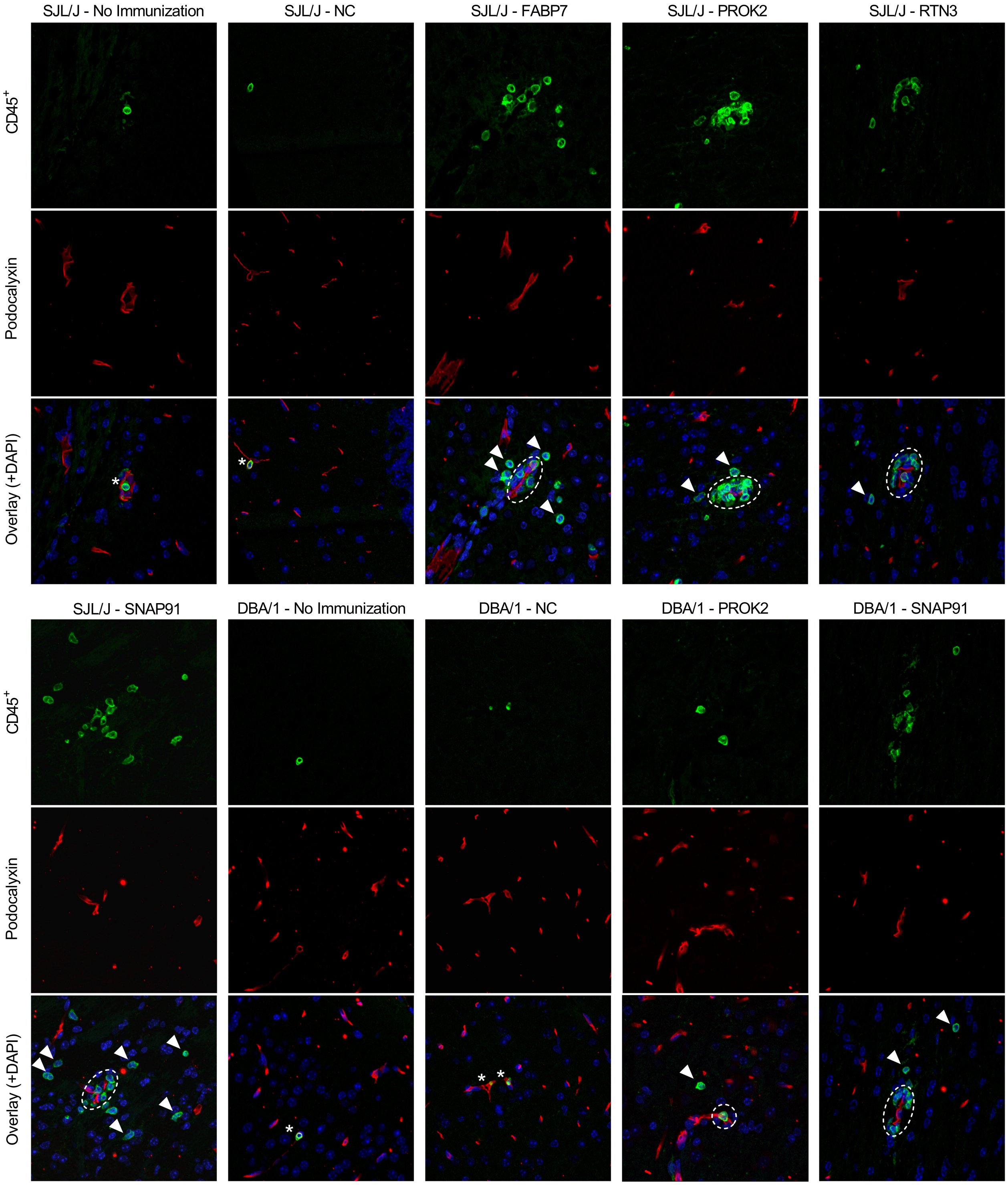

Fig. S7. Location of CNS-infiltrating leukocytes. Representative immunofluorescence of infiltrating leukocytes in the brain of control or autoantigen immunized mice. Top image shows leukocytes ($CD45^+$, green), middle image blood vessels (Podocalyxin, red) and the bottom image the overlaid image together with cell nuclei (DAPI, blue). Arrowheads indicate intraparenchymal leukocytes, dotted white circles indicate perivascular leukocytes and asterixis indicate intravascular leukocytes.

Table S1. Autoantigen screening panel

Protein	PrEST	Pool #
MBP	HPRR2850726	1
MBP	HPRR2850727	1
MAG	HPRR4220035	2
CNP	HPRR2500893	3
CNP	HPRR2500894	3
CNP	HPRR2500895	3
CNP	HPRR2500896	3
CRYAB	HPRR2700063	4
CRYAB	HPRR3880249	4
S100B	HPRR2190015	2
OMG	HPRR1920061	5
OMG	HPRR1920062	5
MOG	HPRR2110197	6
MOBP	HPRR2960632	7
MOBP	HPRR4000195	7
PLP1	HPRR4180637	8
ENO1, ENO2, ENO3	HPRR3360053	8
ALDOC	HPRR4180083	8
GFAP	HPRR350056	9
GFAP	HPRR3830174	9
GFAP	HPRR3830175	9
NFASC	HPRR1920078	10
NFASC	HPRR4190813	10
PEBP1	HPRR1920014	11
PEBP1	HPRR4160717	11
CLDN11	HPRR2010004	12
CLDN11	HPRR2010005	12
CLDN11	HPRR4160179	12
ASCL1	HPRR2840074	13
A1BG	HPRR3140512	13
A1BG	HPRR3140513	13
AFMID	HPRR2501281	14
AFMID	HPRR2501282	14
AFMID	HPRR2501283	14
AFMID	HPRR2501284	14
AMPH	HPRR2440103	15
AMPH	HPRR2440105	15
AMPH	HPRR2440106	15
AQP4	HPRR2140209	16
AQP4	HPRR2140210	16
AQP4	HPRR4040261	16
BCAN	HPRR1920066	17
BCAN	HPRR4180961	17
CYB561D2	HPRR3460137	18
FABP7	HPRR2060033	18
FABP7	HPRR2060034	18
FABP7	HPRR3970332	18
ICAM5	HPRR1950330	19
ICAM5	HPRR1950331	19
MAP2	HPRR1920010	20
MAP2	HPRR1920011	20
MAP6	HPRR3050726	20
MAP6	HPRR3050727	20
MAP6	HPRR3050728	20
MAPK10, 8, 9	HPRR2900006	21
MAPK10	HPRR3730021	21
MT3	HPRR3440018	21
MT3	HPRR3920296	21
NEURL	HPRR3140302	22
NEURL	HPRR3140303	22
NEURL	HPRR3140304	22
NOVA2	HPRR3610021	23

Protein	PrEST	Pool #
PROK2	HPRR3420049	23
PROK2	HPRR3420050	23
PTPRZ1	HPRR1920026	24
PTPRZ1	HPRR1920027	24
PTPRZ1	HPRR4170025	24
RAB3A	HPRR3440020	25
RP11-35N6.1	HPRR2180045	25
RP11-35N6.1	HPRR2180046	25
RP11-35N6.1	HPRR2990130	25
RTN3	HPRR2060024	26
RTN3	HPRR2060025	26
SDK2	HPRR1950089	26
SDK2	HPRR4160333	26
SH3GL2	HPRR2470124	27
SH3GL2	HPRR4110124	27
SLC1A2	HPRR1920041	28
SLC1A2	HPRR4180078	28
SLC1A3	HPRR3010202	28
SLC1A3	HPRR3010204	28
SNAP25	HPRR4180848	29
SNAP91	HPRR2570095	29
SNAP91	HPRR2570096	29
SNAP91	HPRR2570097	29
SNAP91	HPRR2570098	29
SNCB	HPRR3010074	30
STMN3	HPRR1510042	30
STX1A	HPRR2870039	30
TMOD2	HPRR3100160	30
TRIM2	HPRR3000109	31
TRIM2	HPRR3000110	31
TRIM2	HPRR3000111	31
UNC80	HPRR3400083	32
UNC80	HPRR3400086	32
UNC80	HPRR3400088	32
WBSCR17	HPRR1952128	33
WBSCR17	HPRR1952129	33
HSPA4L	HPRR3000429	33
HSPA4L	HPRR3000431	33
B3GNT6	HPRR1952320	34
B3GNT6	HPRR3051098	34
MOAP1	HPRR140345	34
CD9	HPRR3720106	35
CD9	HPRR4110045	35
TSPAN2	HPRR2050163	36
TSPAN2	HPRR4220588	36
PLLP	HPRR3150044	36
MAL	HPRR3460522	37
MAL	HPRR3761244	37
CNTN1	HPRR3140066	38
CNTN1	HPRR3140067	38
INA	HPRR1350076	39
NEFH	HPRR221451	40
NEFH	HPRR4160999	40
NEFM	HPRR2540100	41
NEFM	HPRR2540101	41
NEFM	HPRR2540103	41
CD46	HPRR1950624	42
CD46	HPRR4180423	42
CD55	HPRR280170	43
CD59	HPRR2700037	44
KCNJ10	HPRR4030424	45
KCNJ10	HPRR4030425	45

Table S2. Cohort characteristics

	Screening cohort		1st Validation		2nd Validation		
	MS-Nat (RRMS) (n=16)	HCs (n=9)	MS-Nat (RRMS) (n=61)	HCs (n=28)	MS-Un (RRMS) (n=31)	HCs (n=20)	OND (n=19)
Age (years)	42.1 ± 5.8 (32-53)	40.7 ± 11.1 (24-57)	36.3 ± 9.7 (17-61)	33.9 ± 8.2 (20-49)	34.2 ± 9.7 (19-58)	33.3 ± 6.8 (25-49)	29.3 ± 10.5 (18-57)
Female sex (n)	7 (38.9%)	5 (55.6%)	44 (72.1%)	21 (75.0%)	23 (74.2%)	15 (75.0%)	9 (47.4%)
HLA-DRB1*15:01 (n)	10 (62.5%)	Missing	28 / 56 (50.0%)	7 / 22 (31.8%)	11 / 23 (47.8%)	3 / 12 (25%)	10 / 13* (76.9 %)
HLA-A*02:01 (n)	8 (50.0%)	Missing	24 / 56 (42.9%)	14 / 22 (63.6%)	10 / 23 (43.5%)	7 / 12 (58.3%)	Missing
EDSS (score)	2.5 ± 1.6 (1–5.5)	-	2.1 ± 1.5 (0.0–7.5)	-	1.5 ± 1.3 (0.0–6.5)	-	-
Disease Duration (years)	11.1 ± 5.2 (2.5–20.7)	-	8.9 ± 5.4 (1-20)	-	2.1 ± 3.1 (0.0–12.7)	-	-

± denotes SD. Range or percentage of whole in brackets. n / N denotes the number of individuals for which data was available if it was not the whole cohort. *DR15-status in two cases based on the high linkage disequilibrium with DQB1*06:02. RRMS: Relapsing-remitting MS. HCs: Healthy Controls. Nat: Natalizumab-treated. Un: Untreated. EDSS: Expanded Disability Status Scale.

Table S3. Full-length autoantigen panel

Antigen	Function	Protein description	UniProt ID	Endotoxin wash
Anti-CD3	Positive Control – Polyclonal T cell activation	Monoclonal CD3-2 antibody, Mouse IgG2a isotype.	N/A	N/A
NC	Negative Control – Tag for production	Albumin binding domain – Derived from Strep. Protein G. (69)	N/A	0.1 M NaOH
CMV	Positive Control – Cytomegalovirus epitopes	Fusion protein of 5 epitopes from PP65. (70)	N/A	0.75 M NaOH
FABP7	Novel Candidate Autoantigen	Fatty Acid Binding Protein 7 – Isoform 2$^{1-166}$	O15540-2	0.5 M NaOH
PROK2	Novel Candidate Autoantigen	Prokineticin-2 – Isoform 1$^{28-129}$	Q9HC23-1	1 M NaOH
		Prokineticin-2 – Isoform 2$^{28-108}$	Q9HC23-2	2 M NaOH + 1% Triton X-100
RTN3	Novel Candidate Autoantigen	Reticulon-3$^{1-300}$	O95197	0.5 M NaOH
		Reticulon-3$^{256-700}$	O95197	2 M NaOH
SNAP91	Novel Candidate Autoantigen	Synaptosome Associated Protein 91$^{1-300}$	O60641	3 M NaOH + 1% Triton X-100
		Synaptosome Associated Protein 91$^{274-495}$	O60641	0.1 M NaOH
		Synaptosome Associated Protein 91$^{466-750}$	O60641	1 M NaOH
		Synaptosome Associated Protein 91$^{750-906}$	O60641	0.1 M NaOH
MOG	Established MS Autoantigen	Myelin Oligodendrocyte Glycoprotein$^{30-247}$	Q16653	2 M NaOH + 1% Triton X-100
MBP	Established MS Autoantigen	Myelin Basic Protein$^{1-304}$	P02686-1	1 M NaOH
PLP	Established MS Autoantigen	Proteolipid Protein$^{37-63+89-151+178-233+261-277}$	P60201	2 M NaOH + 1% Triton X-100

Data S1.

Source and complete statistical data for Fig. 1

Data S2.

Source and complete statistical data for Fig. 2

Data S3.

Source and complete statistical data for Fig. 3

Data S4.

Source and complete statistical data for Fig. 4

Data S5.

Source and complete statistical data for Fig. 5

Data S6.

Source and complete statistical data for Fig. 6

Data S7.

Source and complete statistical data for Fig. 7

Data S8.

Source data for Fig. S1-S6
